# Bioactive Diarylheptanoids from *Alpinia coriandriodora*

**DOI:** 10.1007/s13659-020-00264-y

**Published:** 2020-09-09

**Authors:** Xiao-Li Cheng, Han-Xiang Li, Juan Chen, Ping Wu, Jing-Hua Xue, Zhong-Yu Zhou, Nia-He Xia, Xiao-Yi Wei

**Affiliations:** 1 Key Laboratory of Plant Resources Conservation and Sustainable Utilization, South China Botanical Garden, Chinese Academy of Sciences, Tianhe District, Xingke Road 723, Guangzhou, 510650 People’s Republic of China; 2 Guangdong Provincial Key Laboratory of Digital Botanical Garden, South China Botanical Garden, Chinese Academy of Sciences, Xingke Road 723, Tianhe District, Guangzhou, 510650 People’s Republic of China; 3 School of Life Sciences, University of Chinese Academy of Sciences, Yuquanlu 19A, Beijing, 100049 People’s Republic of China

**Keywords:** *Alpinia coriandriodora*, Diarylheptanoid, Antioxidant, Anti-inflammatory

## Abstract

Eight new diarylheptanoids, coriandralpinins A–H (**1**–**8**), were isolated from the rhizomes of *Alpinia coriandriodora*, an edible plant of the ginger family. Their structures, including the absolute configurations, were established by extensive spectroscopic analysis and ECD calculations. Compounds **1**–**8** have a 1,5-*O*-bridged diarylheptanoid structure featuring polyoxygenated aryl units. When evaluated for intracellular antioxidant activity using *t*-BHP stressed RAW264.7 macrophages, all these compounds scavenged reactive oxygen species (ROS) in a concentration-dependent manner. Compounds **3** and **5** also showed inhibitory activity against NO release in LPS-induced RAW 264.7 cells. Six known flavonols, 7,4′-di-*O*-methylkaempferol, 7-*O*-methylquercetin, 7,4′-di-*O*-methylquercetin, 7,3′,4′-tri-*O*- methylquercetin, kaempferol 3-*O*-*β*-d-(6-*O*-*α*-l-rhamnopyranosyl)glucopyranoside, and 3-*O*-*β*-d-glucopyranuronosylquercetin were also isolated and characterized from the rhizomes.

**Electronic supplementary material:**

The online version of this article (10.1007/s13659-020-00264-y) contains supplementary material, which is available to authorized users.

## Introduction

Metabolism of oxygen is crucial to life for the production of energy to support biological process. As a consequence of the aerobic metabolism, reactive oxygen species (ROS) are continuously generated in all living organisms, and controlled by several antioxidant mechanisms [1, 2]. However, overproduction and/or mismanagement of ROS may evade cellular antioxidant defense systems, resulting in the general phenomenon of oxidative stress. The redox imbalance leads to cellular damage, which is implicated with inflammatory process and various chronic degeneration diseases such as cancer, cardiovascular disease and diabetes [3–6]. In recent years there has been increasing interest in antioxidants of plant origin [7–10], particularly those from edible plants, and many of these naturally occurring compounds have been shown to possess protective efficacies against oxidative stress and inflammation-related diseases [11–14].

**Fig. 1:**
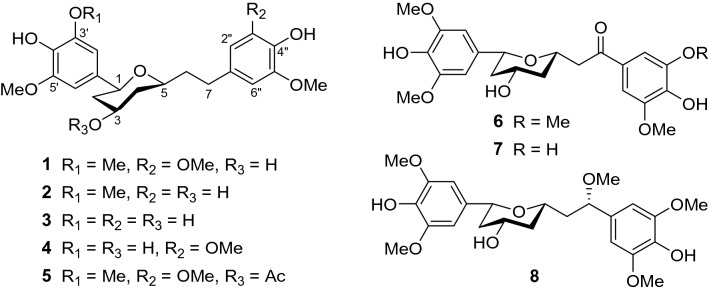
Structures of coriandralpinins A–G (**1**–**8**)

As part of our going effort to isolate and identify antioxidant and anti-inflammatory compounds from edible and medicinal plants, our attention was drawn to *Alpinia coriandriodora* D. Fang (Zigiberaceae), a perennial herb distributed in Guangxi, China. Plants of the genus *Alpinia* are rich in diarylheptanoids, sesquiterpenoids, and monoterpenes, many of which possess antioxidant [15, 16], anti-inflammatory [17], hepatoprotective [18], and anticancer activities [19, 20]. Although *A. coriandriodora* has long been used by local inhabitants as food spices, few chemical investigations on the species have been reported so far. We now present herein the isolation, characterization, and biological activities of eight new diarylheptanoids, coriandralpinins A–G (**1**–**8**) (Fig. 1), from the rhizomes of this plant. The isolation of six known flavonoids is also described.

## Results and Discussion

The powder of dry *A. coriandriodora* rhizomes was extracted with EtOH. The crude extract was suspended in water and extracted successively with petroleum ether, EtOAc, and *n*-BuOH. The resultant three fractions were separately subjected to column chromatography over silica gel, ODS, and Sephadex LH-20, followed by semi-preparative HPLC, to afford new diarylheptanoids **1**−**8** (Fig. 1) and known flavonols **9**−**14** (Fig. S1, Supplementary Material).

**Fig. 2:**
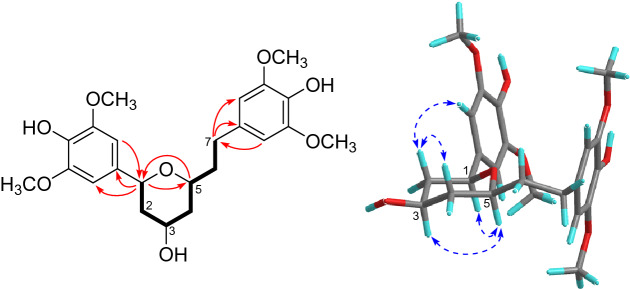
COSY (bold lines) and key HMBC (arrows) and NOESY (dashed arrows) correlations of **1** (the 3D conformer represents the DFT global energy minimum)

**Fig. 3:**
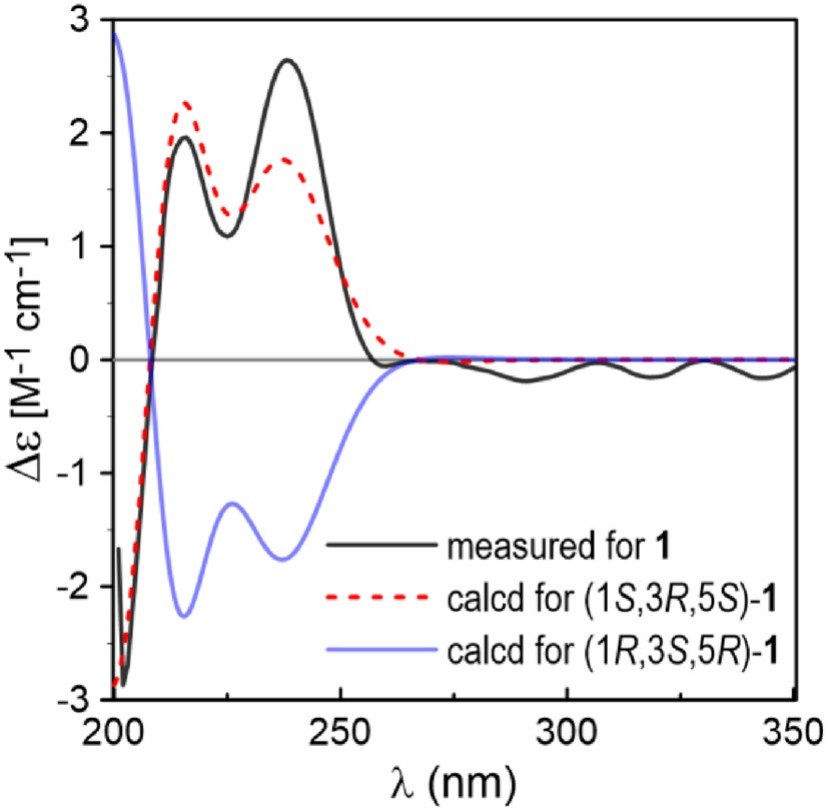
Comparison between the measured and PBE0/Def2-SVP/PCM (MeOH) calculated ECD spectra of **1** (σ = 0.34, shift =  + 11 nm)

Coriandralpinin A (**1**) was obtained as light brown oil. Its HRESIMS showed a pseudo-molecular ion at *m*/*z* 435.2009 ([M + H]^+^), indicating the molecular formula C_23_H_30_O_8_. The ^13^C NMR spectrum contained signals for 23 carbons including twelve aromatic (four methine and eight non-protonated), four methoxy, four aliphatic methylene, and three aliphatic methine carbons. Combined analysis of the ^1^H and ^13^C NMR data (Tables 1 and 2) with COSY and HSQC spectra revealed the presence of two 3,5-dimethoxy-4-hydroxyphenyl moieties [*δ*_H_ 6.41, 6.63 (each 2H, s), 3.84, 3.90 (each 6H, s); *δ*_C_ 103.0, 105.2, 56.4, 56.5] and a seven membered aliphatic chain (Fig. 2). In the HMBC spectrum (Fig. 2), correlations of H-1 with C-1′, -2′, and -6′ and of H_2_-7 with C-1′', -2′', and -6′' suggested that the two phenyl rings were connected to the alkyl chain terminal carbons C-1 and C-7, respectively. In addition, correlations of H-1/C-5 and H-5/C-1 indicated that C-1 was linked to C-5 via an oxygen atom to form a tetrahydropyran unit. The relative stereochemistry of **1** was elucidated on the basis of proton coupling constants and NOESY correlations. In the ^1^H NMR spectrum, H-1 (*δ*_H_ 4.26) and H-2ax (*δ*_H_ 1.50) appeared as a double doublet (*J* = 11.4, 2.0 Hz) and an apparent quartet (*J* = 11.4 Hz), respectively, suggesting that H-1 and H-3 (*δ*_H_ 3.94) are both in axial positions. The remaining oxymethine proton H-5 was also assigned to be axial by the NOE interactions observed for H-5/H-1 and H-5/H-3 (Fig. 2). Based on the established relative configuration, the absolute configuration of **1** was assigned by electronic circular dichroism (ECD) spectrum aided with ECD/TDDFT calculations. The experimental ECD spectrum of **1** exhibited positive Cotton effects at 242 and 216 nm, which matched well with those of the theoretical spectrum calculated for the 1*S*,3*R*,5*S* isomer (Fig. 3). On the basis of the above analysis, the structure of **1** was established as (1*S*,3*R*,5*S*)-1,5-epoxy-1,7-bis(4-hydroxy-3,5-dimethoxyphenyl)heptan-3-ol.

**Fig. 4:**
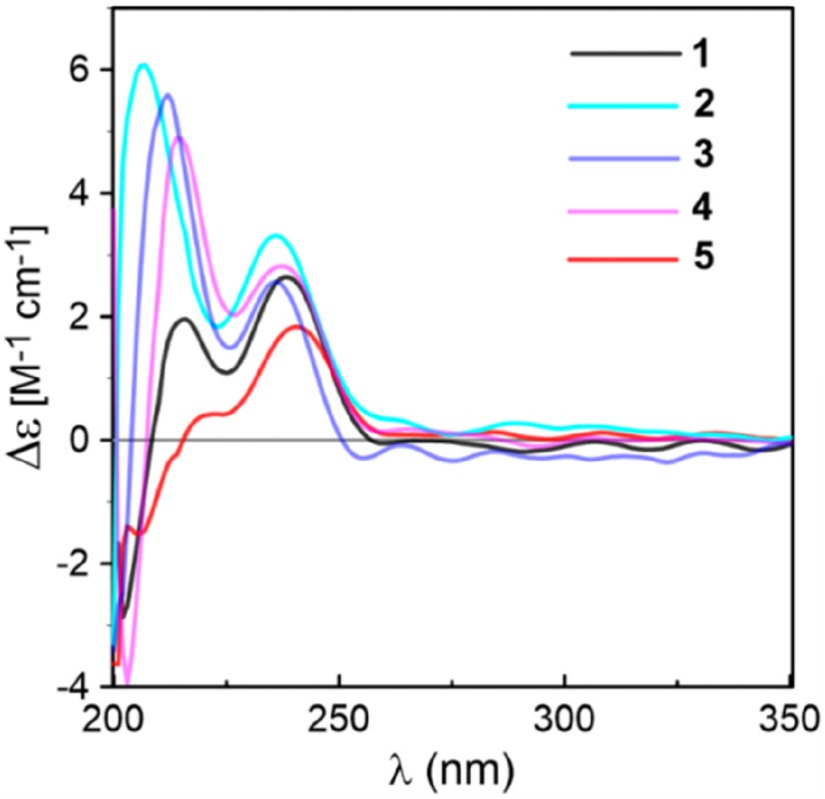
Comparison of the ECD spectra measured for **1**–**5**

Coriandralpinins B–E (**2**–**5**) were elucidated to have diarylheptanoid structures closely related to coriandralpinin A (**1**) on the basis of their UV, NMR (Tables 1 and 2), and HRESIMS data. Compound **2** was determined to have a molecular formula containing one CH_2_O unit less than **1**. It differed from **1** in that the 3,5-dimethoxy-4-hydroxyphenyl group at C-7 in **1** was replaced by a 4-hydroxy-3-methoxyphenyl group as indicated by an ABX spin system at *δ*_H_ 6.82 (1H, d, *J* = 8.0 Hz), 6.68 (1H, dd, *J* = 8.0, 1.9 Hz), and 6.69 (1H, d, *J* = 1.9 Hz) for three aromatic protons and a 3H singlet at *δ*_H_ 3.83 for an aromatic methoxy in the ^1^H NMR spectrum. Compounds **3** had a molecular formula with one CH_2_ unit less than **2**. Its ^1^H NMR spectrum was closely similar to that of **2** except that signals for the C-1 aryl group appeared as two meta-coupled doublets at *δ*_H_ 6.54 and 6.53 (each 1H, *J* = 2.0 Hz) and a 3H singlet at *δ*_H_ 3.84, indicating a 3,4-dihydroxy-5-methoxyphenyl group. The molecular formula of compound **4** was determined to be C_22_H_28_O_8_, consisting of a CH_2_ unit less than **1**. Its 1D NMR data for the phenyl group at C-7 were similar to those of **1** and the resonances for the C-1 phenyl group were almost identical with those of **3** (Tables 1 and 2), indicating a 3′-*O*-demethyl derivative of **1**. Compound **5** had a molecular formula of C_25_H_32_O_9_, requiring an additional C_2_H_2_O unit compared with that of **1**. Its 1D NMR spectra differed from those of **1** in the presence of additional signals [*δ*_H_ 2.06 (3H, s); *δ*_C_ 21.4, 170.8] for an acetyl group, which was attached to C-3 via an ester linkage as indicated by the downfield shift of H-3 (*δ*_H_ 5.02), suggesting a 3-*O*-acetyl derivative of **1**. The above structures deduced for **2**–**5** were also supported by COSY, HSQC, and HMBC spectra. Their stereochemistries were all assigned to be the same as that of **1** on the basis of ^1^H NMR coupling constants and NOESY correlations as well as by the similarity of their ECD spectra with that of **1** (Fig. 4). Therefore, the structures of these compound were elucidated as (1*S*,3*R*,5*S*)-1,5-epoxy-1-(4-hydroxy-3,5-dimethoxyphenyl)-7-(4-hydroxy-3-methoxyphenyl)heptan-3-ol (**2**), (1*S*,3*R*,5*S*)-1,5-epoxy-1-(3,4-dihydroxy-5-methoxyphenyl)-7-(4-hydroxy-3-methoxyphenyl)heptan-3-ol (**3**), (1*S*,3*R*,5*S*)-1,5-epoxy-1-(3,4-dihydroxy-5-methoxyphenyl)-7-(4-hydroxy-3,5-dimethoxyphenyl)heptan-3-ol (**4**), (1*S*,3*R*,5*S*)-3-*O*-aceyl-1,5-epoxy-1,7-bis(4-hydroxy-3,5-dimethoxyphenyl)heptan-3-ol (**5**). It is noted that two diarylheptanoids of unknown absolute configurations isolated from ginger [21] had been reported to possess the same planar and relative stereochemical structures as **2** and **3** and they had optical rotations ([α]_D_) with values similar but sign opposite to those of **2** and **3**, indicating the reported compounds are likely the enantiomers of our compounds.

**Fig. 5:**
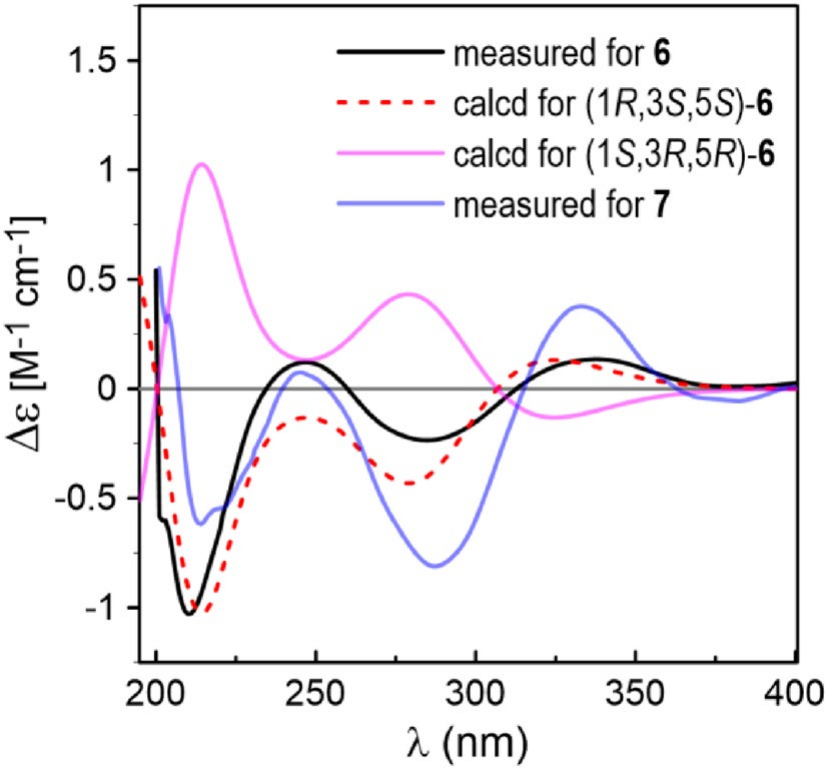
Comparison of the measured ECD spectra of **6** and **7** with PBE0/TZVP/PCM(MeOH) calculated ECD spectra of (1*R*,3*S*,5*S*)- and (1*S*,3*R*,5*R*)-**6** (σ = 0.38, shift = −11 nm)

Coriandralpinin F (**6**) had a molecular formula of C_23_H_28_O_9_ according to HRESIMS data. Its UV spectrum was largely similar to those of **1**–**5** but differed in that the absorption maximum at the longest wavelength was significantly red-shifted (301 nm vs 271–280 nm), suggesting the presence of a chromophore with extended conjugated system. This finding was consistent with the ^1^H and ^13^C NMR data (Tables 1 and 2), which were comparable to those of **1** except the carbon signal indicating the presence of a conjugated ketone carbonyl (*δ*_C_ 199.5) and the absence of proton and carbon resonances for C-7 methylene. Detailed analysis of the 1D and 2D NMR data readily derived a structure of 7-keto derivative of **1**, including the relative stereochemistry. As the experimental ECD spectrum of **6** was significantly different from the spectrum of **1**, theoretical computations of ECD spectrum were carried out in order to solve the absolute stereochemistry. As a result (Fig. 5), chiral carbons in **6** were shown to be inverted relative to those in **1**–**5**, designating this compound as 1*R*,3*S*,5*S*. Coriandralpinin G (**7**) was a 3′'-*O*-demethyl derivative of **6** as elucidated by analysis of the 1D NMR (Tables 1 and 2), 2D NMR, and HRESIMS data. Its absolute stereochemistry was assigned to be the same as that of **6** based on the nearly parallel experimental ECD curves of the two compounds (Fig. 5). Thus, the structures of compounds **6** and **7** were established to be (1*R*,3*S*,5*S*)-1,5-epoxy-3-hydroxy-1,7-bis(4-hydroxy-3,5-dimethoxyphenyl)heptan-7-one and (1*R*,3*S*,5*S*)-1,5-epoxy-3-hydroxy-1-(4-hydroxy-3,5-dimethoxyphenyl)-7-(3,4-dihydroxy-5-methoxyphenyl)heptan-7-one, respectively.

**Fig. 6:**
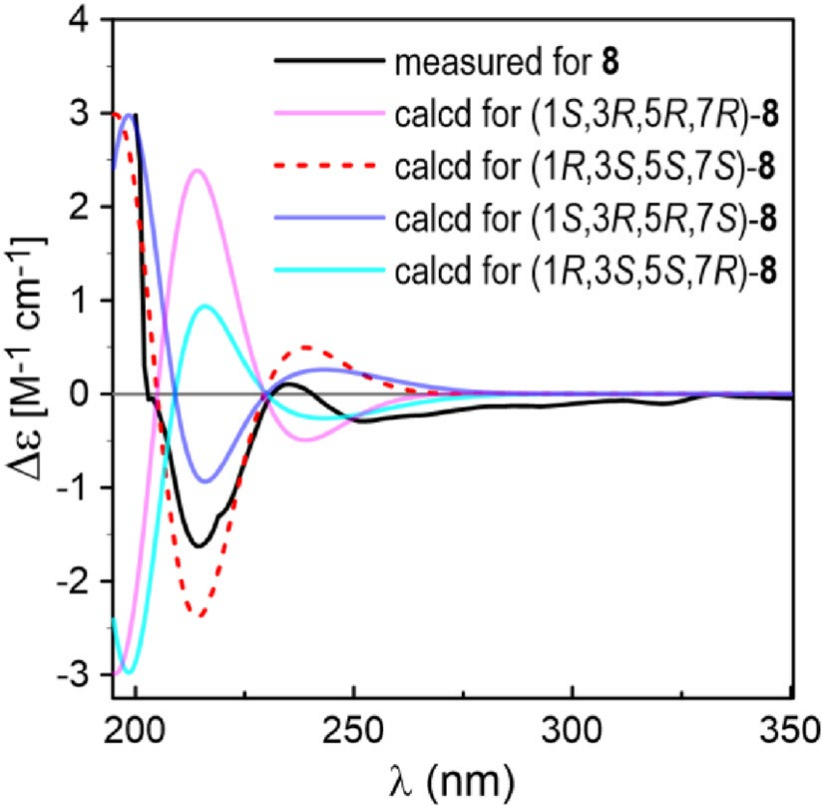
Comparison between the ECD spectra measured for **8** and PBE0/TZVP/PCM calculated for four possible stereoisomers of **8** (σ = 0.38, shift = −2 nm)

**Fig. 7:**
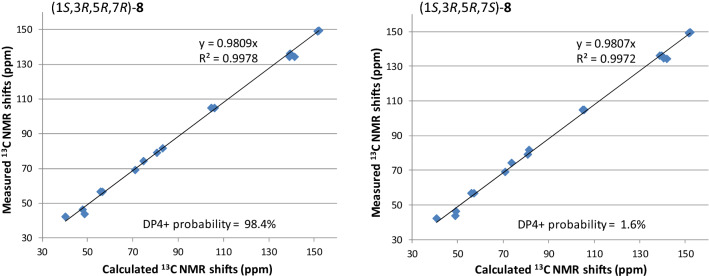
Linear regression analysis of the calculated ^13^C NMR shifts of two possible stereoisomers against the measured shifts of **8** and the DP4 + probability for **8** to be the stereoisomers

The planar structure of the last diarylheptanoid, coriandralpinin H (**8**), was established to be 7-methoxylated **1** by analysis of NMR and MS data. Different from the other analogues, compound **8** had one more chiral center C-7, which brought a challenge of solving its stereochemistry. Analysis of proton coupling constants (Table 1) and NOESY data readily assigned the relative configuration of tetrahydropyran ring to be the same as that in **1**–**7**, but gave no clear information for the configuration at C-7. In order to assign the absolute configuration of **8**, ECD/TDDFT calculations were carried out for 1*S*,3*R*,5*R*,7*R* and 1*S*,3*R*,5*R*,7*S* stereoisomers. Unfortunately, the simulated ECD spectrum of (1*S*,3*R*,5*R*,7*S*)-**8** and the mirror-image of the calculated spectrum for (1*S*,3*R*,5*R*,7*R*)-**8** were both in good agreement with the measured spectrum of **8** (Fig. 6). To further define the absolute configuration, ^13^C NMR shifts of (1*S*,3*R*,5*R*,7*R*)-**8** and (1*S*,3*R*,5*R*,7*S*)-**8** were calculated using the gauge including atomic orbitals (GIAO) method at the mPW1PW91/6–311 + G(d,p)/PCM level of theory. The calculated ^13^C NMR data of (1*S*,3*R*,5*R*,7*R*)-**8** showed a better match with the measured data of **8** (DP4 + probability: 98.4%) than (1*S*,3*R*,5*R*,7*S*)-**8** (DP4 + probability: 1.6%) (Fig. 7), suggesting a 1*S*,3*R*,5*R*,7*R* or 1*R*,3*S*,5*S*,7*S* configuration. This, together with the results of ECD simulations, allowed designation of **8** as 1*R*,3*S*,5*S*,7*S*. In consequence, the structure of **8** was determined as (1*R*,3*S*,5*S*,7*S*)-1,5-epoxy-7-methoxy-1,7-bis(4-hydroxy-3,5-dimethoxyphenyl)heptan-3-ol.

The isolated known flavonoids were identified to be 7,4′-di-*O*-methylkaempferol (**9**) [22], 7-*O*-methylquercetin (**10**) [23], 7,4′-di-*O*-methylquercetin (**11**) [24], 7,3′,4′-tri-*O*-methyl-quercetin (**12**) [25], kaempferol 3-*O*-*β*-d-(6-*O*-α-l-rhamnopyranosyl)glucopyranoside (**13**) [26], and 3-*O*-*β*-d-glucopyranuronosylquercetin (**14**) [27] (Fig. S1, Supplementary Material) by interpretation of their spectroscopic data as well as by comparison of the data with reported values in literatures.

**Fig. 8:**
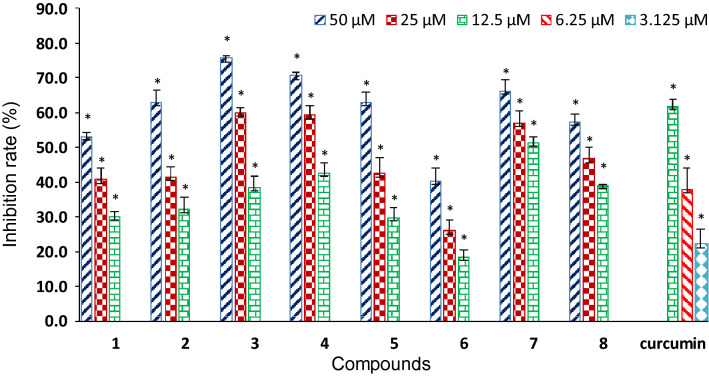
Concentration-dependent inhibition of *t*-BHP-induced ROS production in RAW264.7 cells by compounds **1**–**8** and the positive control curcumin (^*^P < 0.05 vs *t*-BHP alone)

Compounds **1**–**8** were evaluated for antioxidant and anti-inflammatory activities in respect that the two activities are of the most common and interesting biological properties of diarylheptanoids [28–30]. The antioxidant activity evaluation was conducted using a more physiologically relevant method, the cellular antioxidant activity (CAA) assay [29, 30], with RAW 264.7 macrophages as the test cells. The concentrations of compounds used in the assay were 50 μM and lowers according to cell viability assay by MTT method, which showed that **1**–**8** had no observable toxicity to RAW 264.7 cells at the concentration up to 100 μM. As indicated in Fig. 8 and Table 3, all these compounds were capable of significantly decreasing *t*-BHP-induced ROS production in RAW 264.7 cells in a concentration-dependent manner (Fig. 8), although none of them was more potent than the positive control curcumin. Among them, 3′- or 3′'-OH bearing **3**, **4**, and **7** exhibited better activity (IC_50_: 12.0–18.0 μM) than their corresponding 3′- or 3′'-*O*-methyl derivatives **2**, **5**, and **6** (Table 3). Besides, the change of 7-keto carbonyl to the methoxylated methine was shown to increase the activity (**6** vs **8**).

**Table 1:** ^1^H NMR (500 MHz) data for compounds **1**–**8**

position	**1**^*a*^	**2**^*a*^	**3**^*b*^	**4**^*b*^	**5**^*a*^	**6**^*b*^	**7**^*b*^	**8**^*b*^
1	4.26 dd (11.4, 2.0)	4.26 dd (11.4, 2.0)	4.19 dd (11.4, 1.9)	4.18 dd (11.4, 1.9)	4.33 dd (11.5, 2.0)	4.34 dd (11.4, 1.9)	4.32 d (10.1)	4.30–4.36 m
2	ax 1.50 q (11.4) eq 2.20 ddt (12.5, 4.3, 2.0)	ax 1.51 q (11.4) eq 2.20 ddt (12.5, 4.3, 2.0)	ax 1.41 q (11.4) eq 2.08 ddt (12.5, 4.3, 2.0)	ax 1.41 q (11.4) eq 2.08 ddt (12.4, 4.2, 1.9)	ax 1.59 q (11.7) eq 2.23 br d (12.7, 4.1, 1.8)	ax 1.36–1.46 m eq 2.11–2.17 m	ax 1.34–1.44 m eq 2.10–2.18 m	ax 1.46 q (11.6) eq 2.13 ddt (12.4, 4.0, 1.6)
3	3.94 m	3.95 m	3.81 m	3.82 m	5.02 m	3.93 m	3.95 m	3.90 m
4	ax 1.33 q (11.4) eq 2.03 ddt (12.2, 4.1, 1.8)	ax 1.33 q (11.4) eq 2.03 ddt (12.3, 4.1, 1.9)	ax 1.23 q (11.5) eq 1.95 ddt (12.4, 4.4, 1.9)	ax 1.23 q (11.3) eq 1.94 ddt (12.3, 4.3, 1.9)	ax 1.44 q (11.6) eq 2.09 m	ax 1.36–1.46 m eq 2.11–2.17 m	ax 1.34–1.44 m eq 2.10–2.18 m	ax 1.24 q (11.6) eq 2.01 ddt (12.4, 4.0, 1.7)
5	3.45 m	3.45 m	3.42 m	3.40 m	3.51 m	4.10 m	4.10 m	3.76 m
6	1.98 m 1.81 m	1.98 m 1.80 m	1.87 m 1.75 m	1.75 m 1.87 m	1.99 m 1.81 m	3.36 dd (15.2, 7.1) 3.08 dd (15.2, 5.0)	3.35 dd (15.1, 7.1) 2.98 dd (15.1, 5.2)	1.93 m 1.83 m
7	2.67–2.75 m	2.64–2.77 m	2.61–2.72 m	2.61–2.71 m	2.61–2.73 m			4.30–4.36 m
2′	6.63 s	6.62 s	6.54 d (2.0)	6.55 d (1.9)	6.61 s	6.54 s	6.54 s	6.67 s
6′	6.63 s	6.62 s	6.53 d (2.0)	6.53 d (1.9)	6.61 s	6.54 s	6.54 s	6.67 s
2″	6.41 s	6.69 d (1.9)	6.76 d (2.0)	6.45 s	6.40 s	7.36 s	7.23 d (2.0)	6.58 s
3″		6.82 d (8.0)	6.69 d (8.0)					
6″	6.41 s	6.68 dd (8.0,1.9)	6.62 dd (8.0, 2.0)	6.45 s	6.40 s	7.36 s	7.20 d (2.0)	6.58 s
7-OCH_3_								3.19 s
3′-OCH_3_	3.90 s	3.90 s			3.90 s	3.85 s	3.75 s	3.85 s
5′-OCH_3_	3.90 s	3.90 s	3.84 s	3.83 s	3.90 s	3.85 s	3.75 s	3.85 s
3″-OCH_3_	3.84 s			3.77 s	3.84 s	3.74 s		3.81 s
5″-OCH_3_	3.84 s	3.83 s	3.78 s	3.77 s	3.84 s	3.74 s	3.84 s	3.81 s
CH_3_CO					2.06 s			

^a^CDCl_3_ used as solvent

^b^CD_3_OD used as solvent

**Table 2:** ^13^C NMR (125 MHz) data for compounds **1**–**8**

Position	**1**^*a*^	**2**^*a*^	**3**^*b*^	**4**^*b*^	**5**^*a*^	**6**^*b*^	**7**^*b*^	**8**^*b*^
1	77.7	77.6	79.0	79.0	77.6	79.1	78.9	79.2
2	43.1	42.9	43.7	43.8	39.3	43.6	43.7	43.7
3	68.6	68.7	69.0	69.0	70.9	68.7	68.7	69.0
4	41.0	40.9	41.9	41.8	37.1	41.7	41.8	42.3
5	75.0	75.0	76.2	76.2	75.0	74.7	74.7	74.3
6	37.9	37.9	39.1	39.0	37.9	45.3	45.2	46.2
7	32.0	31.5	32.3	32.8	32.0	199.5	199.6	81.8
1'	133.4	133.4	134.6	134.5	133.0	134.4	134.4	134.5
2'	103.0	103.1	108.0	108.0	103.0	104.5	104.3	104.8
3'	147.1	147.1	146.4	146.4	147.1	148.9	149.0	149.1
4'	134.3	134.0	134.5	134.2	134.3	136.5	135.8	136.0
5'	147.1	147.1	149.5	149.4	147.1	148.9	149.0	149.1
6'	103.0	103.1	102.8	102.7	103.0	104.5	104.3	104.8
1′'	133.2	134.3	135.0	134.5	133.1	129.7	129.8	134.3
2′'	105.2	111.2	113.3	106.7	105.2	107.8	111.6	104.8
3′'	147.1	114.3	116.1	149.1	147.1	149.0	146.3	149.4
4′'	132.9	143.8	145.5	134.6	133.0	142.6	141.2	135.9
5′'	147.1	146.5	148.8	149.1	147.1	149.0	149.2	149.4
6′'	105.2	121.1	121.8	106.7	105.2	107.8	105.3	104.8
7-OCH_3_								56.7
3′-OCH_3_	56.5	56.5			56.5	56.8	56.7	56.8
5′-OCH_3_	56.5	56.5	56.6	56.6	56.5	56.8	56.7	56.8
3′'-OCH_3_	56.4			56.7	56.4	56.7		56.7
5′'-OCH_3_	56.4	56.0	56.3	56.7	56.4	56.7	56.7	56.7
CH_3_*C*O					170.8			
*C*H_3_CO					21.4			

^a^CDCl_3_ used as solvent

^b^CD_3_OD used as solvent

**Table 3:** Inhibitory activity of compounds **1**–**8** against *t*-BHP-induced ROS and LPS-induced NO production in RAW264.7 cells

compound	Inhibition of ROS (IC_50_, μM)^a^	Inhibition of NO (IC_50_, μM)^a^
**1**	43.0 ± 1.5	> 50
**2**	31.1 ± 2.6	> 50
**3**	18.0 ± 0.7	36.9 ± 1.1
**4**	17.6 ± 1.0	> 50
**5**	31.0 ± 3.3	34.1 ± 0.6
**6**	> 50	> 50
**7**	12.0 ± 2.1	> 50
**8**	29.9 ± 1.8	> 50
Curcumin	8.6 ± 0.4	
Dexamethasone		33.6 ± 1.5

^a^Data are presented as mean ± SD (*n* = 3)

The potential anti-inflammatory activity of compounds **1**–**8** was evaluated by measuring their inhibitory effects on nitric oxide (NO) production in lipopolysaccharide (LPS)-induced RAW 264.7 cells (Table 3). Among these diarylheptanoids, compounds **3** and **5** exhibited inhibitory activity against NO release with IC_50_ values of 36.9 and 34.1 μM, respectively, which were comparable to that of the positive drug control dexamethasone (IC_50_: 33.6 μM). However, other isolated analogues were inactive in the assay (IC_50_ > 50 μM).

In summary, phytochemical analysis of *A. Coriandriodora* rhizomes led to the characterization of a group of unique 1,5-*O*-bridged diarylheptanoids, coriandralpinins A–H (**1**–**8**). These compounds exhibited remarkable intracellular antioxidant activity in concentration-dependent manner. Compounds **3** and **5** also inhibited NO release in LPS-induced RAW 264.7 cells. The results suggested that the edible plant *A. coriandriodora* is a potential source for dietary supplements.

## Experimental Section

### General Experimental Procedures

Optical rotations were obtained on a Perkin-Elmer 341 polarimeter with MeOH as solvent. UV and ECD spectra were recorded in MeOH with a Chirascan CD spectrometer (Applied Photophysics LTD., England). ^1^H NMR, ^13^C NMR, and 2D NMR spectra were recorded in chloroform-*d* or methanol-*d*_4_ on a Brucker AVIII 500 M instrument for ^1^H at 500 and for ^13^C at 125 MHz using the residual solvent peak as reference. High-resolution electrospray ionization mass spectrometry (HRESIMS) was obtained on Bruker maXis Q-TOF mass spectrometer. ESIMS data were obtained on an MDS SCIEX API 2000 LC/MS instrument (SCIEX, Toronto, Canada). HRESIMS data were obtained on a Bruker Bio TOF IIIQ mass spectrometer (Bruker Daltonics, Billerica, MA, U.S.A.). Preparative HPLC was performed with an HPLC system equipped with a Shimadzu LC-6AD pump (Shimadzu, Kyoto, Japan) using a YMC-pack ODS-A column (5 μm, 10 × 250 mm, YMC, Kyoto, Japan). For column chromatography, silica gel 60 (100–200 mesh, Qingdao Marine Chemical Ltd., Qingdao, China) and YMC ODS (75 μm, YMC, Kyoto, Japan) were used. TLC was performed using HSGF254 silica gel plates (Yantai Jiangyou Silica Gel Development Co. Ltd., Yantai, China).

### Plant Material

The rhizomes of *A. Coriandriodora* were purchased from Nanning, Guangxi, China, in January 2018. A voucher specimen (specimen number SC-42–3-7) was deposited at the Herbarium of South China Botanical Garden, Chinese Academy of Sciences.

### Extraction and Isolation

The fresh rhisomes of *A. Coriandriodora* (1.55 kg), after being dried at 50 °C in an oven, were crushed into powder (260 g) and then extracted with 95% EtOH for 48 h (× 3). The crude extract (58 g) was suspended in water and extracted successively with petroleum ether (PE), EtOAc, and *n*-BuOH for three times each. The PE-soluble fraction (13 g) was subjected to silica gel column chromatography (CC) using gradient PE-acetone mixtures (v/v, 100:0–60:40) to obtain ten fractions (Fr. A1 to Fr. A10). Fr. A5 (470 mg) was separated by Sephadex LH-20 CC using PE-CH_2_Cl_2_-MeOH (1:1:1) to afford compound **9** (150 mg). Fr. A10 was applied to ODS CC using MeOH-H_2_O (v/v, 30:70–100:0) to give ten subfractions (Fr. A10-1 to Fr. A10-10). Fr. A10-9 (12 mg) and Fr. A10-10 (30 mg) were further separated by Sephadex LH-20 CC using PE-CH_2_Cl_2_-MeOH (1:1:1) to obtain **11** (3 mg) and **12** (25 mg), respectively. Fr. A10-5 (6 mg) was purified by preparative HPLC using 30% MeCN to afford **10** (1 mg, *t*_R_ = 25.6 min). The EtOAc-soluble fraction (11.42 g) was subjected to silica gel CC, eluted with gradient CH_2_Cl_2_-MeOH mixtures (v/v, 100:0 to 50:50), to afford eleven fractions (Fr. B1 to Fr. B11). Fr. B5 (271 mg) was chromatographed on a Sephadex LH-20 column using PE-CH_2_Cl_2_-MeOH (1:1:1) to yield **1** (244 mg). Fr. B6 (1.17 g) was further applied to ODS CC using MeOH-H_2_O (v/v, 1:9 to 10:0) to yield eleven subfractions (Fr. B6-1 to Fr. B6-11). Fr. B6-4 was subjected to Sephadex LH-20 CC using PE-CH_2_Cl_2_-MeOH (1:1:1) followed by preparative HPLC to yield **6** (2 mg, *t*_R_ = 36.6 min) and **2** (9 mg, *t*_R_ = 95.0 min) using 20% CH_3_CN and **5** (5 mg, *t*_R_ = 79.6 min) using 34% CH_3_CN. Fr. B8 (3.42 g) was subjected to ODS CC using MeOH-H_2_O (v/v, 1:9 to 10:0) to give twelve subfractions (Fr. B8-1 to Fr. B8-12). Fr. B8-4 was further separated by HPLC using 17% CH_3_CN to afford **7** (10 mg, *t*_R_ = 27.1 min). Fr. B8-5 was subjected to Sephadex LH-20 CC using PE-CH_2_Cl_2_-MeOH (1:1:1) followed by preparative HPLC using 20% CH_3_CN to yield **8** (3 mg, *t*_R_ = 51.1 min), **4** (76 mg, *t*_R_ = 45.3 min), and **3** (11 mg, *t*_R_ = 54.1 min). The *n*-BuOH-soluble fraction (1 g) was subjected ODS CC using MeOH-H_2_O (v/v, 1:9–3:7) to give three fractions (Fr. C1 to Fr. C3). Fr. C1 (141 mg) was separated by preparative HPLC using 22% MeCN to afford **13** (22 mg, *t*_R_ = 11.2 min) and **14** (8 mg, *t*_R_ = 13.0 min).

**Coriandralpinin A (1)**: light brown oil; [*α*]_D_^20^ + 51.1 (*c* 0.33, MeOH); UV (MeOH) λ_max_ nm (log *ε*): 206 (3.93), 230 (3.12), 272 (2.30); CD (MeOH) Δ*ε* 219 (+ 1.97), 239 (+ 2.63); ^1^H and ^13^C NMR data were depicted Tables 1 and 2. HRESIMS *m/z* 435.2009 [M + H]^+^ (calcd for C_23_H_31_O_8_, 435.2014), *m/z* 457.1836 [M + Na]^+^ (calcd for C_23_H_30_NaO_8_, 457.1833).

**Coriandralpinin B (2)**: light brown oil, [*α*]_D_^20^ + 54.5 (*c* 0.70, MeOH). UV (MeOH) λ_max_ nm (log *ε*): 202 (3.96), 230 (3.20), 280 (2.72); CD (MeOH) Δ*ε* 207 (+ 6.07), 236 (+ 3.31); ^1^H and ^13^C NMR data were depicted Tables 1 and 2. ( +)-HRESIMS: *m/z* 405.1914 [M + H]^+^ (calcd for C_22_H_29_O_7_, 405.1908), *m/z* 427.1736 [M + Na]^+^ (calcd for C_22_H_28_NaO_7_, 427.1728).

**Coriandralpinin C (3)**: light yellow oil, [*α*]_D_^20^ + 54.9 (*c* 0.99, MeOH). UV (MeOH) λ_max_ nm (log *ε*): 201 (4.00), 230 (3.24), 279 (2.71); CD (MeOH) Δ*ε* 212 (+ 5.59), 236 (+ 2.57); ^1^H and ^13^C NMR data were depicted Tables 1 and 2. ( +)-HRESIMS: *m/z* 391.1748 [M + H]^+^ (calcd for C_21_H_27_O_7_, 391.1752), *m/z* 413.1571 [M + Na]^+^ (calcd for C_21_H_26_NaO_7_, 413.1571).

**Coriandralpinin D (4)**: orange oil, [*α*]_D_^20^ + 56.4 (*c* 0.25, MeOH). UV (MeOH) λ_max_ nm (log *ε*): 205 (3.97), 229 (3.18), 271 (2.31); CD (MeOH) Δ*ε* 214 (+ 4.89), 237 (+ 2.82); ^1^H and ^13^C NMR data were depicted Tables 1 and 2. ( +)-HRESIMS: *m/z* 421.1851 [M + H]^+^ (calcd for C_22_H_29_O_8_, 421.1857), *m/z* 443.1674 [M + Na]^+^ (calcd for C_22_H_28_NaO_8_, 443.1677).

Coriandralpinin E (5): light yellow oil, [*α*]_D_^20^ + 34.8 (*c* 0.56, MeOH). UV (MeOH) λ_max_ nm (log *ε*): 206 (3.90), 230 (3.10), 271 (2.30); CD (MeOH) Δ*ε* 223 (+ 0.42), 241 (+ 1.83); ^1^H and ^13^C NMR data were depicted Tables 1 and 2. ( +)-HRESIMS: *m/z* 477.2114 [M + H]^+^ (calcd for C_25_H_33_O_9_, 477.2120), *m/z* 499.1934 [M + Na]^+^ (calcd for C_25_H_32_NaO_9_, 499.1939).

Coriandralpinin F (6): light brown oil, [*α*]_D_^20^ + 10.3 (*c* 0.18, MeOH). UV (MeOH) λ_max_ nm (log *ε*): 206 (3.47), 225 (3.06), 301 (2.72); CD (MeOH) Δ*ε* 210 (−  1.03), 285 (− 0.24), 338 (+ 0.14); ^1^H and ^13^C NMR data were depicted Tables 1 and 2. ( +)-HRESIMS: *m/z* 449.1804 [M + H]^+^ (calcd for C_23_H_29_O_9_, 449.1807), *m/z* 471.1623 [M + Na]^+^ (calcd for C_23_H_28_NaO_9_, 471.1626).

**Coriandralpinin G (7)**: yellow oil, [*α*]_D_^20^ + 19.8 (*c* 0.81, MeOH). UV (MeOH) λ_max_ nm (log *ε*): 206 (3.77), 225 (3.33), 301 (2.72); CD (MeOH) Δ*ε* 214 (-0.62), 287 (− 0.81), 333 (+ 0.38); ^1^H and ^13^C NMR data were depicted Tables 1 and 2. ( +)-HRESIMS: *m/z* 435.1658 [M + H]^+^ (calcd for C_22_H_27_O_9_, 435.1650), *m/z* 457.1466 [M + Na]^+^ (calcd for C_22_H_26_NaO_9_, 457.1470).

**Coriandralpinin H (8)**: light brown oil, [*α*]_D_^20^ + 34.9 (*c* 0.25, MeOH). UV (MeOH) λ_max_ nm (log *ε*): 206 (3.92), 235 (3.10), 271 (2.31); ^1^H and ^13^C NMR data were depicted Tables 1 and 2. ( +)-HRESIMS: *m/z* 487.1942 [M + Na]^+^ (calcd for C_24_H_32_NaO_9_, 487.1939), *m/z* 951.3994 [2 M + Na]^+^ (calcd for C_48_H_64_NaO_18_, 951.3985).

### Cell Viability Assay

The murine macrophage RAW 264.7 cell line was obtained from Kunming Institute of Zoology, Chinese Academy of Sciences (Kunming, China). The cells were cultured in DMEM medium supplemented with 10% heated-inactivated fetal bovine serum in a 37 °C, 5% CO_2_ incubator. Compounds **1**–**8**, curcumin, and *t*-BHP were assessed for cytotoxicity against RAW 264.7 cells at various concentrations (100, 50, 10, and 2 μM) by MTT method as previously described [31].

### Cellular Antioxidant Activity Assay

In the MTT cell viability assay, 50 µM of *t*-BHP (Sigma-Aldrich, USA, TBH70X) treatment for 3 h provoked about 60% of cell death and this condition was selected for the subsequent experiments as reported [29, 30]. Briefly, RAW 264.7 cells (4 × 10^4^ cells/well) were seeded into a black 96-well plate and allowed to growth for 24 h, then treated with either compounds (100, 50, 25, 12.5, and 6.25 µM) or the positive control curcumin (20, 10, 5, 2.5, and 1.25 µM) for 24 h. After stained with 20 μM DCFH-DA (Sigma, USA) for 1 h in darkness, the cells were exposed to 50 μM *t*-BHP for 1 h to induce ROS generation. The supernatants of cell cultures were measured for intracellular ROS levels using the fluorescence intensity at excitation wavelength of 485 nm and the emission wavelength of 530 nm by microplate reader (Tecan Group Ltd., Swizerland).

### Anti-Inflammatory Activity Evaluation

The in vitro anti-inflammatory activity was evaluated by measuring the inhibitory effects of compounds on NO production in LPS-stimulated RAW 264.7 macrophages as previously described [32]. Briefly, the supernatants of cells were obtained as described above and RAW 264.7 cells were pretreated with different concentrations of compounds **1**–**8** (50, 25, 12.5, 6.25, and 3.125 μM) at 37 °C for 1 h, followed by stimulated with LPS (0.1 µg/ml) for 24 h. DMSO (0.1%) and dexamethasone (50, 25, 12.5, 6.25 and 3.125 μM) were used as vehicle and positive controls, respectively. The levels of NO were determined using commercial NO assay kit (Beyotime Institute of Biotechnology, China). 50 μL of Griess reagent I and 50 μL of Griess reagent II were added to 100 μL of the supernatants of cells (9 × 10^5^/mL).

### Computational Methods

Molecular Merck force field (MMFF) and DFT/TDDFT calculations were performed with Spartan'14 software package (Wavefunction Inc., Irvine, CA, USA) and Gaussian09 program package [33], respectively. The structures, (1*S*,3*R*,5*S*)-**1**, (1*S*,3*R*,5*R*)-**6**, (1*S*,3*R*,5*R*,7*R*)-**8**, and (1*S*,3*R*,5*R*,7*S*)-**8**, were applied to the theoretical calculations. MMFF conformational search generated low-energy conformers within a 10 kcal/mol energy window were subjected to geometry optimization using DFT method at the B3LYP/def2-SVP [34] level of theory with the solvent model PCM for MeOH. Frequency calculations were run at the same level to verify that each optimized conformer was a true minimum and to estimate their relative thermal free energies (Δ*G*). The optimized minima (Table S1, Supplementary Material) within the relative energies of 4.0 kcal/mol were subjected to the higher level of energy calculations at the M06-2X [35]/def2-TZVP [34] level with the solvent model SMD for MeOH. The TDDFT calculations were performed using the hybrid PBE0 (PBE1PBE) [36, 37] and M06-2X functionals, and Ahlrichs’ basis sets def2-SVP and TZVP [38] with the PCM for MeOH. The number of excited states per each molecule was 42 for all compounds. The ECD spectra were generated by the program SpecDis [39] using a Gaussian band shape from rotational strengths. The final calculated spectra were generated by averaging calculated spectra of the low-energy conformers according to the Boltzmann weighting of each conformer in MeOH solution. The theoretical ECD spectra of (1*R*,3*S*,5*R*)-**1**, (1*R*,3*S*,5*S*)-**6**, (1*R*,3*S*,5*S*,7*S*)-**8**, and (1*R*,3*S*,5*S*,7*R*)-**8**, were obtained by mirror-inversion of the calculated spectra for (1*S*,3*R*,5*S*)-**1**, (1*S*,3*R*,5*R*)-**6**, (1*S*,3*R*,5*R*,7*R*)-**8**, and (1*S*,3*R*,6*R*,7*S*)-**8**, respectively.

For calculations of the ^13^C NMR shifts of **8**, low-energy conformers of the stereoisomers, (1*S*,3*R*,5*R*,7*R*)-**8** and (1*S*,3*R*,5*R*,7*S*)-**8**, obtained in above ECD simulations, were subjected to NMR calculations using the gauge including atomic orbitals (GIAO) method [40, 41, 42] at the mPW1PW91/6–311 + G(d,p)/PCM level [43]. The unscaled chemical shifts (*δ*_u_) were computed using TMS as reference standard according to *δ*_u_ = σ_0_ − σ^x^ (where σ^x^ is the Boltzmann averaged shielding tensor and σ_0_ is the shielding tensor of TMS computed at the same level employed for σ^x^). The Boltzmann averaging was done at 298.15 K using the relative energies obtained from the single-point NMR calculations [44, 45]. The goodness of fit between the predicted ^13^C NMR data of the two stereoisomers and the experimental shifts of compound **8** were evaluated by the improved DP4 probability (DP4 +) [44, 45].

## Electronic supplementary material

Below is the link to the electronic supplementary material.

Supplementary file1 (PDF 8915 kb)
