# Supplementary material for: Bioactive Diarylheptanoids from Alpinia coriandriodora
Source: Nat Prod Bioprospect. 2020 Sep 9;11(1):63–72. doi: 10.1007/s13659-020-00264-y (PMC7933350; doi:10.1007/s13659-020-00264-y)
Supplement: Supplementary file 1 — Supplementary file1 (PDF 8915 kb) [file 13659_2020_264_MOESM1_ESM.pdf]

Supporting Information

Bioactive Diarylheptanoids from *Alpinia coriandriodora*

Xiao-Li Cheng[1,3], Han-Xiang Li[1,2], Juan Chen[1,2], Ping Wu[1,2,3], Jing-Hua Xue[1,2], Zhong-Yu Zhou[1,2,3], Nia-He Xia[1,2,3], Xiao-Yi Wei[1,2,3]

[1] Key Laboratory of Plant Resources Conservation and Sustainable Utilization, South China Botanical Garden, Chinese Academy of Sciences, Guangzhou 510650, China

[2] Guangdong Provincial Key Laboratory of Digital Botanical Garden, South China Botanical Garden, Chinese Academy of Sciences, Guangzhou 510650, China

[3] School of Life Sciences, University of Chinese Academy of Sciences, Yuquanlu 19A, Beijing 100049, China

Contents

Table S1. Calculated relative thermal and free energies and equilibrium populations of low-energy conformers of compounds **1**, **6**, and **8**. 1

Table S2. Calculated ^{13}C NMR data of two possible stereoisomers of **8** and their goodness of fit with the measured shifts of **8**........ 2

Fig. S1 Structures of compounds **9**–**14** 3

Fig. S2 ^{1}H NMR spectrum of compound **1**. 4

Fig. S3 ^{13}C NMR spectrum of compound **1**. 4

Fig. S4 ^{1}H–^{1}H COSY spectrum of compound **1**. 5

Fig. S5 HSQC spectrum of compound **1**. 5

Fig. S6 HMBC spectrum of compound **1**. 6

Fig. S7 NOESY spectrum of compound **1**. 6

Fig. S8 HRESI-MS spectrum of compound **1**. 7

Fig. S9 ^{1}H NMR spectrum of compound **2**. 8

Fig. S10 ^{13}C NMR spectrum of compound **2**. 8

Fig. S11 HRESI-MS spectrum of compound **2**. 9

Fig. S12 ^{1}H NMR spectrum of compound **3**. 10

Fig. S13 ^{13}C NMR spectrum of compound **3**. 10

Fig. S14 HRESI-MS spectrum of compound **3**. 11

Fig. S15 ^{1}H NMR spectrum of compound **4**. 12

Fig. S16 ^{13}C NMR spectrum of compound **4**. 12

Fig. S17 ^{1}H–^{1}H COSY spectrum of compound **4**. 13

Fig. S18 HSQC spectrum of compound **4**. 13

Fig. S19 HMBC spectrum of compound **4**. 14

Fig. S20 NOESY spectrum of compound **4**. 14

Fig. S21 HRESI-MS spectrum of compound **4**. 15

Fig. S22 ^{1}H NMR spectrum of compound **5**. 16

Fig. S23 ^{13}C NMR spectrum of compound **5**. 16

Fig. S24 ^{1}H–^{1}H COSY spectrum of compound **5**. 17

Fig. S25 HSQC spectrum of compound **5**. 17

Fig. S26 HMBC spectrum of compound **5**. 18

Fig. S27 NOESY spectrum of compound **5**. 18

Fig. S28 HRESI-MS spectrum of compound **5**. 19

Fig. S29 ^{1}H NMR spectrum of compound **6**. 20

Fig. S30 ^{13}C NMR spectrum of compound **6**. 20

Fig. S31 ^{1}H–^{1}H COSY spectrum of compound **6**. 21

Fig. S32 HSQC spectrum of compound **6**. 21

Fig. S33 HMBC spectrum of compound **6**. 22

Fig. S34 NOESY spectrum of compound **6**. 22

Fig. S35 HRESI-MS spectrum of compound **6**. 23

Fig. S36 ^{1}H NMR spectrum of compound **7**. 24

Fig. S37 ^{13}C NMR spectrum of compound **7**. 24

Fig. S38 ^{1}H–^{1}H COSY spectrum of compound **7**. 25

Fig. S39 HSQC spectrum of compound **7**. 25

Fig. S40 HMBC spectrum of compound **7**. 26

Fig. S41 NOESY spectrum of compound **7**. 26

Fig. S42 HRESI-MS spectrum of compound **7**. 27

Fig. S43 ^{1}H NMR spectrum of compound **8**. 28

Fig. S44 ^{13}C NMR spectrum of compound **8**. 28

Fig. S45 ^{1}H–^{1}H COSY spectrum of compound **8**. 29

Fig. S46 HSQC spectrum of compound **8**. 29

Fig. S47 HMBC spectrum of compound **8**. 30

Fig. S48 NOESY spectrum of compound **8**. 30

Fig. S49 HRESI-MS spectrum of compound **8**. 31

Table S1. M06-2X/def2-TZVP/SMD//B3LYP/def2-SVP/PCM calculated relative thermal energies (Δ*E*), relative free energies (Δ*G*), and equilibrium populations (P)[a] of low-energy conformers of compounds **1**, **6**, and **8** in MeOH solution.

conformer	*E* (kcal/mol)	*G* (kcal/mol)	P (%)
(1*S*,3*R*,5*S*)-**1**			
**1a**	0.0	0.0	33.2
**1b**	1.203	0.101	28.0
**1c**	2.222	0.279	20.7
**1d**	2.998	1.103	5.2
**1e**	3.104	1.128	4.9
**1f**	1.261	1.252	4.0
**1g**	2.682	1.480	2.7
**1h** [b]	0.972	1.911	1.3
(1*S*,3*R*,5*R*)-**6**			
**6a**	1.345	0.0	21.9
**6b**	0.0	0.153	16.9
**6c**	0.873	0.507	9.3
**6d**	0.106	0.562	8.5
**6e**	0.292	0.600	7.9
**6f**	0.894	0.626	7.6
**6g**	0.334	0.658	7.2
**6h**	0.424	0.760	6.1
**6i**	1.847	1.138	3.2
**6j**	1.914	1.167	3.1
**6k**	1.803	1.201	2.9
**6l**	1.864	1.221	2.8
**6m**	0.879	1.349	2.2
**6n** [b]	1.764	2.347	0.4
(1*S*,3*R*,5*R*,7*R*)-**8**			
R-**8a**	0.0	0.0	67.9
R-**8b**	1.568	0.836	16.5
R-**8c**	2.342	1.270	7.9
R-**8d**	0.476	1.764	3.4
R-**8e**	2.229	1.990	2.4
R-**8f**	2.705	2.135	1.8
(1*S*,3*R*,5*R*,7*S*)-**8**			
S-**8a**	0.675	0.0	61.2
S-**8b**	0.0	0.749	17.3
S-**8c**	1.755	1.107	9.4
S-**8d**	0.625	1.492	4.9
S-**8e**	2.819	1.703	3.4
S-**8f**	2.804	1.748	3.2

[a] From G values at 298.15 K. [b] Conformer not applied to ECD/TDDFT calculations

Table S2. The mPW1PW91/6-311+G(d,p)/PCM//B3LYP/def2-SVP calculated ^{13}C NMR data for (1*S*,3*R*,5*R*,7*R*)-**8** and (1*S*,3*R*,5*R*,7*S*)-**8** and their goodness of fit with the measured shifts of **8**.

position	(1*S*,3*R*,5*R*,7*R*)-**8**			(1*S*,3*R*,5*R*,7*S*)-**8**			**8**
	σ^x	δ_u	δ_s	σ^x	δ_u	δ_s	δ_{exp}
C-3''	34.71	152.27	149.36	34.94	152.04	149.11	149.4
C-5''	34.71	152.27	149.36	34.94	152.04	149.11	149.4
C-3'	35.32	151.6	148.76	35.25	151.73	148.81	149.1
C-5'	35.32	151.6	148.76	35.25	151.73	148.81	149.1
C-1'	45.57	141.4	138.71	45.01	141.97	139.23	134.5
C-4'	47.56	139.42	136.76	48.17	138.81	136.13	136.0
C-4''	47.62	139.36	136.70	47.40	139.58	136.89	135.9
C-1''	47.92	139.06	136.41	46.36	140.62	137.91	134.3
C-2''	83.20	106.20	104.17	81.34	105.59	103.55	104.8
C-2'	82.34	104.64	102.64	82.11	104.87	102.85	104.8
C-6'	82.34	104.64	102.64	82.11	104.87	102.85	104.8
C-6''	83.20	106.20	104.17	81.341	105.59	103.55	104.8
C-7	103.66	83.32	81.73	105.58	81.41	79.83	81.8
C-1	106.19	80.79	79.25	106.00	80.98	79.41	79.2
C-5	112.35	74.63	73.21	113.20	73.78	72.35	74.3
C-3	115.77	71.21	69.85	116.08	70.90	69.53	69.0
7-OCH_3	130.19	56.79	55.71	129.80	57.18	56.08	56.7
3'- OCH_3	131.20	55.78	54.72	130.85	56.135	55.05	56.8
3''-OCH_3	131.17	55.81	54.75	130.90	56.08	54.99	56.7
5''-OCH_3	131.17	55.81	54.75	130.90	56.08	54.99	56.7
5'- OCH_3	131.20	55.78	54.72	130.85	56.14	55.05	56.8
C-2	138.13	48.85	47.92	138.08	48.90	47.96	43.7
C-6	138.97	48.01	47.09	137.53	49.45	48.50	46.2
C-4	146.57	40.41	39.649	146.32	40.66	39.88	42.3
Probability	sDP4+ = 98.3%<br>uDP4+ = 52.0%<br>DP4+ = 98.4%			sDP4+ = 1.7%<br>uDP4+ = 48.0%<br>DP4+ = 1.6%			

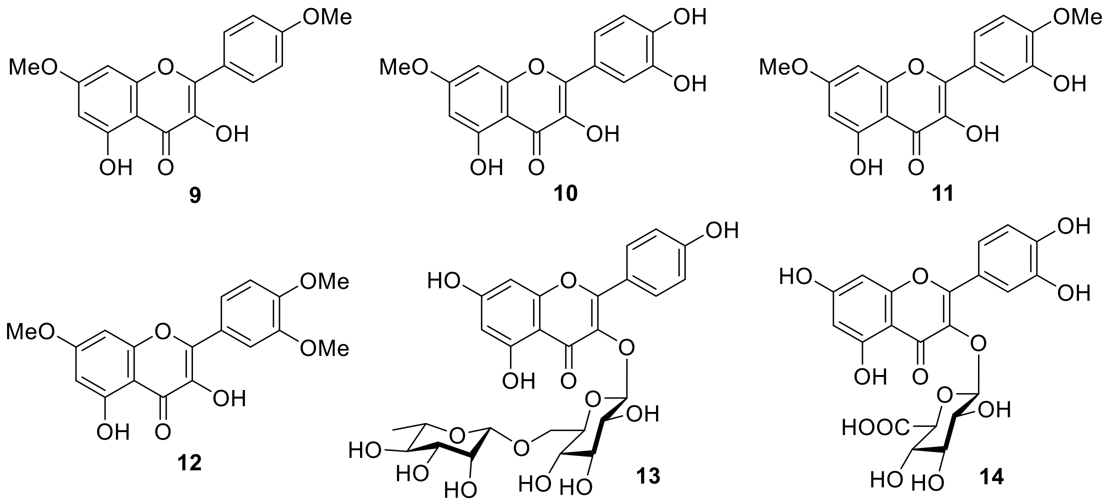

Fig. S1 Structures of compounds **9–14**

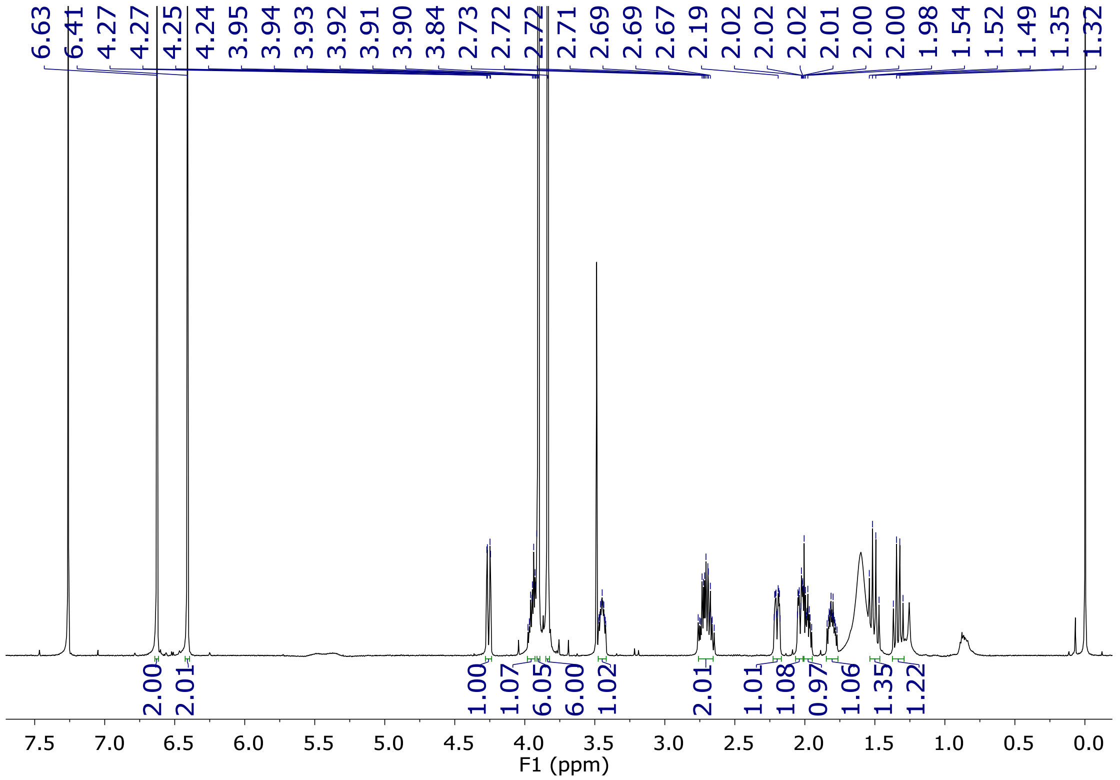

Fig. S2 ^{1}H NMR spectrum of compound **1**.

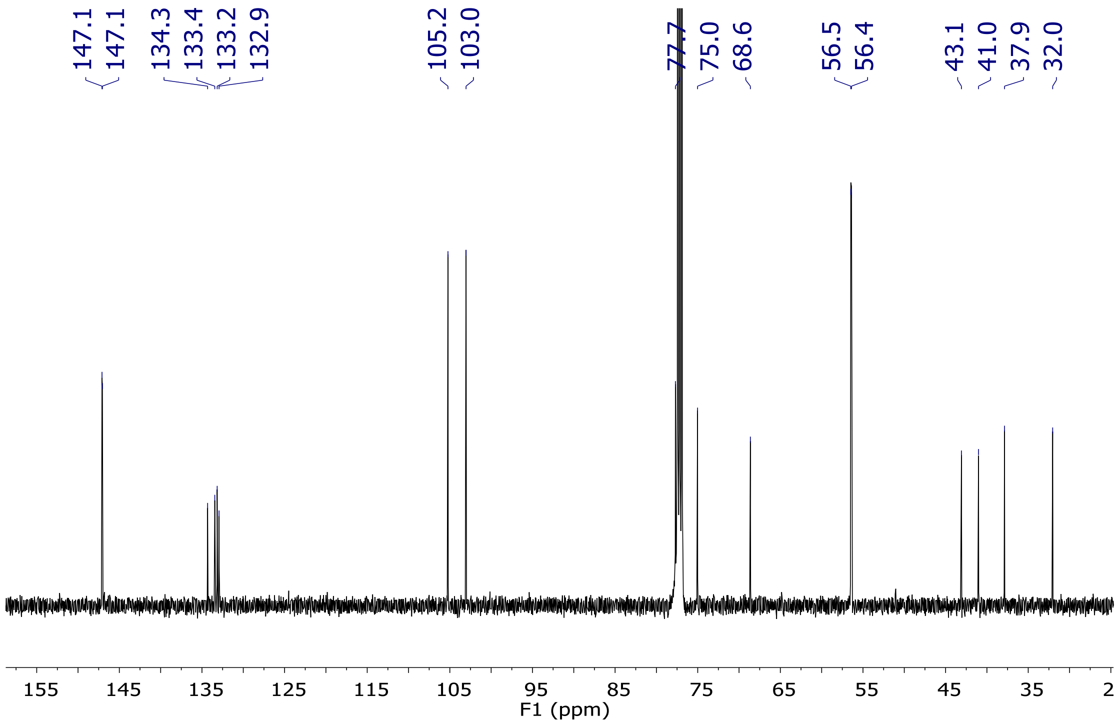

Fig. S3 ^{13}C NMR spectrum of compound **1**.

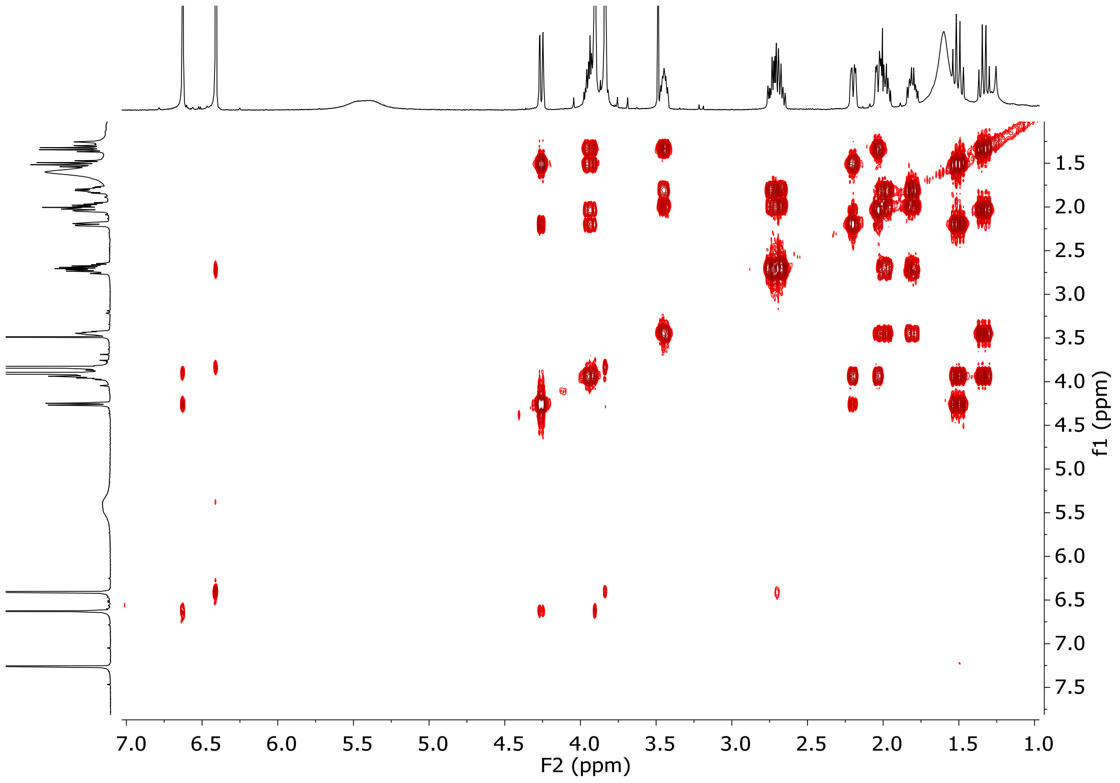

Fig. S4 ^{1}H–^{1}H COSY spectrum of compound **1**.

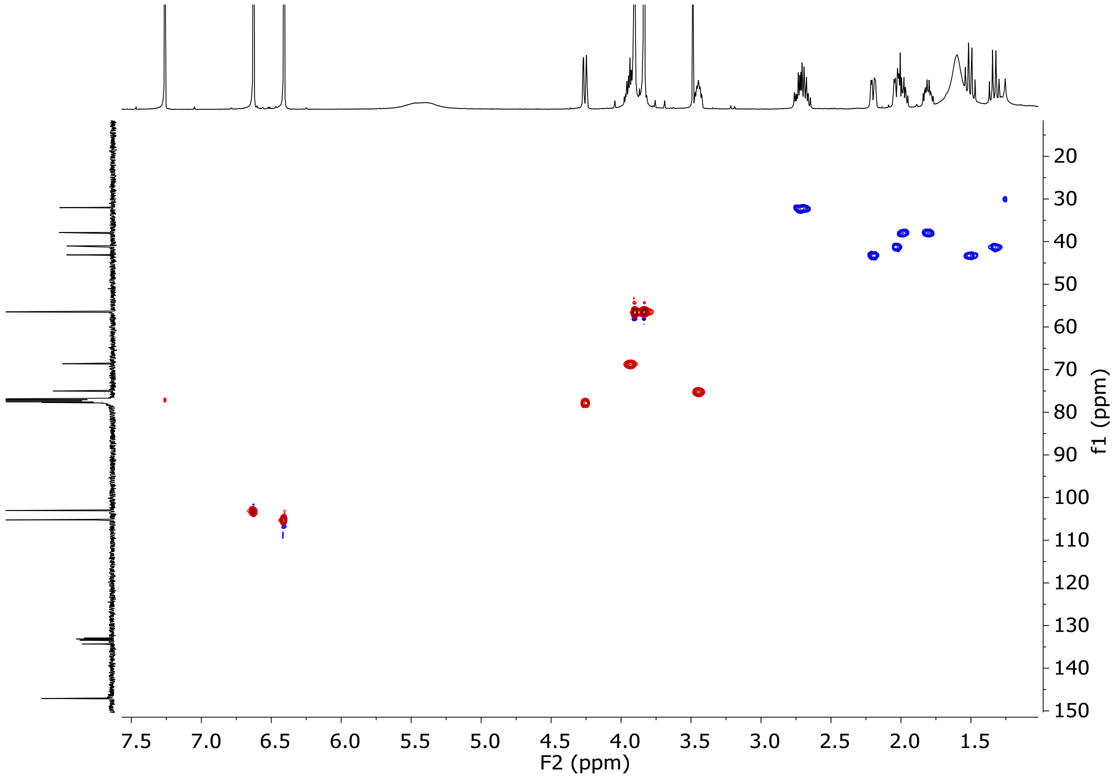

Fig. S5 HSQC spectrum of compound **1**.

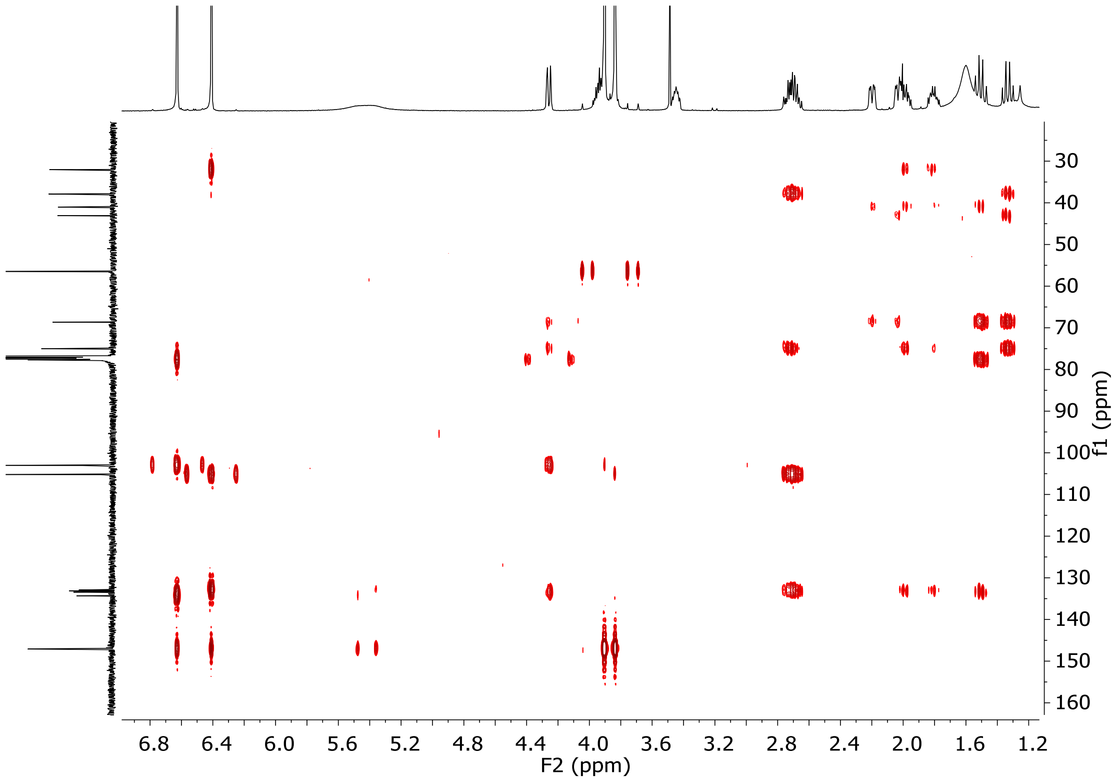

Fig. S6 HMBC spectrum of compound **1**.

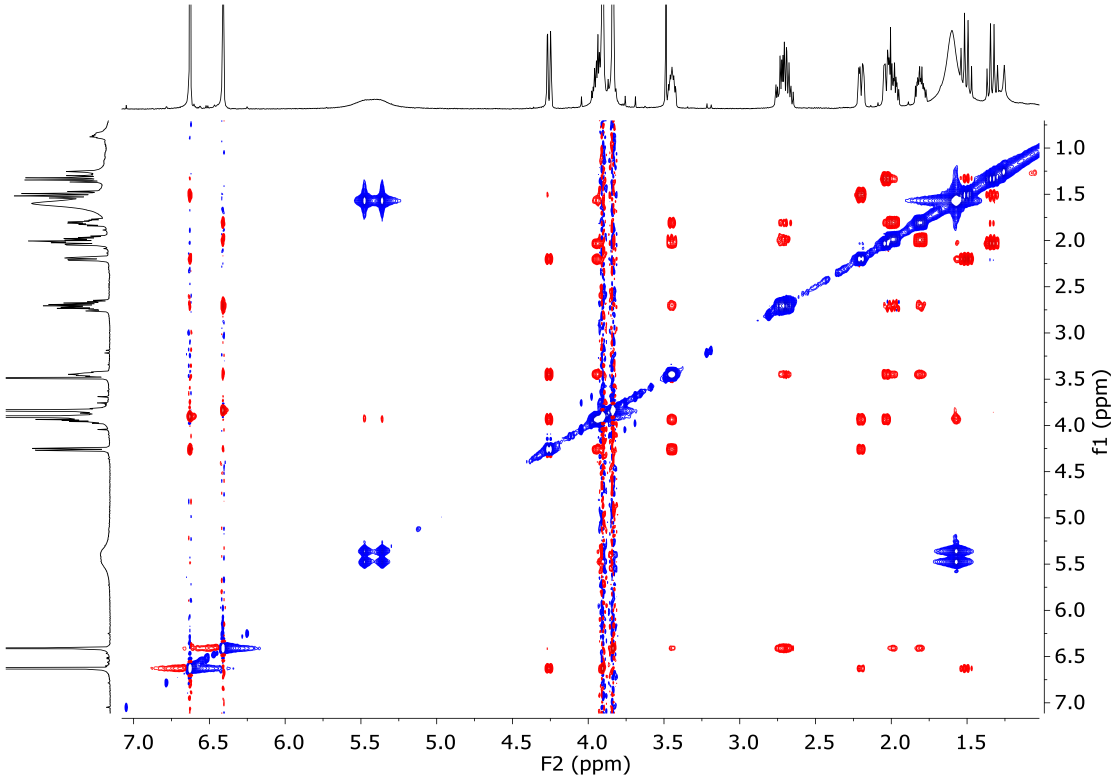

Fig. S7 NOESY spectrum of compound **1**.

Acquisition Parameter

Source Type	ESI	Ion Polarity	Positive	Set Nebulizer	0.4 Bar
Focus	Active	Set Capillary	4500 V	Set Dry Heater	180 °C
Scan Begin	100 m/z	Set End Plate Offset	-500 V	Set Dry Gas	4.0 l/min
Scan End	2000 m/z	Set Charging Voltage	0 V	Set Divert Valve	Waste
		Set Corona	0 nA	Set APCI Heater	0 °C

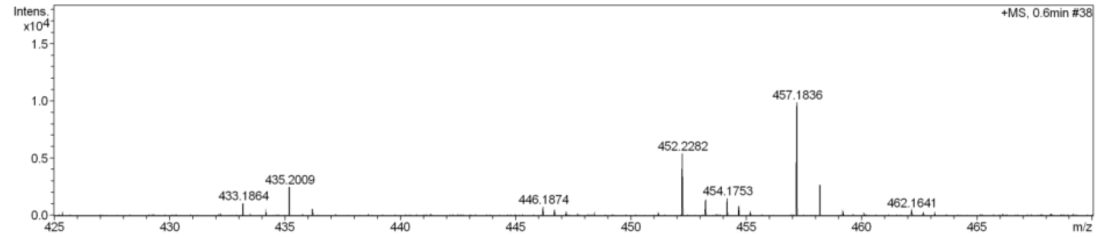

Meas. m/z	#	Ion Formula	Score	m/z	err [ppm]	err [mDa]	mSigma	rdb	e⁻ Conf	N-Rule
435.2009	1	C23H31O8	100.00	435.2013	0.9	0.4	8.4	8.5	even	ok
457.1836	1	C23H30NaO8	100.00	457.1833	0.6	0.3	10.7	8.5	even	ok
869.3954	1	C46H61O16	100.00	869.3954	0.0	0.0	22.2	16.5	even	ok
891.3777	1	C46H60NaO16	100.00	891.3774	-0.4	-0.4	7.1	16.5	even	ok

Fig. S8 HRESI-MS spectrum of compound **1**.

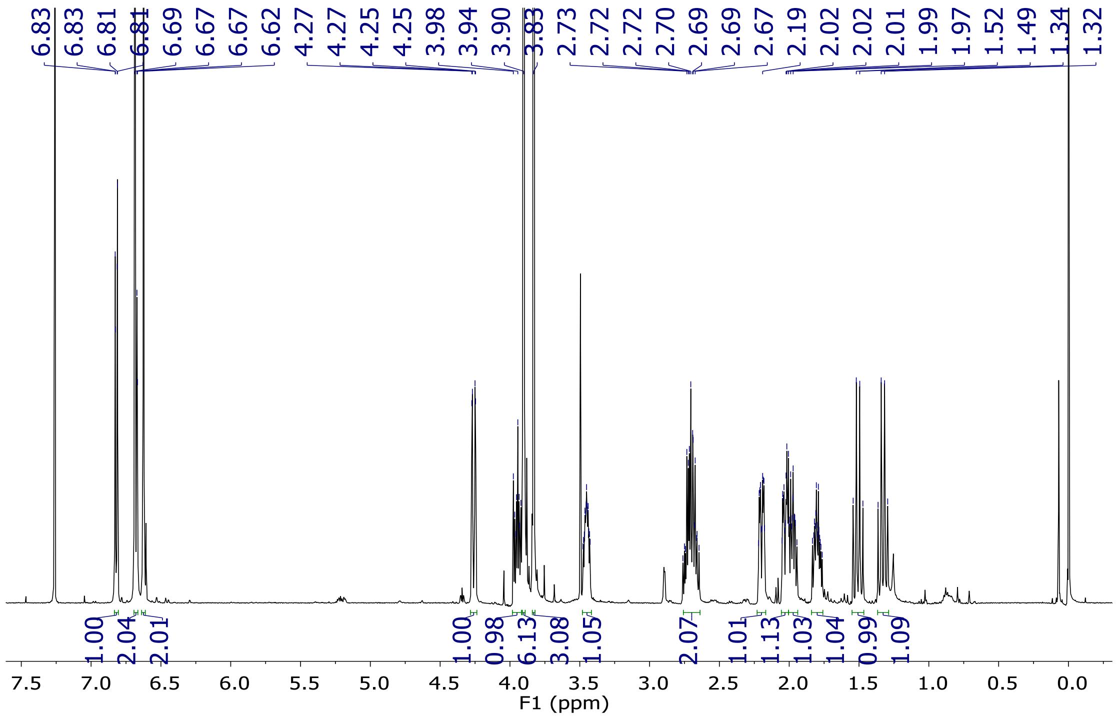

Fig. S9 ^{1}H NMR spectrum of compound **2**.

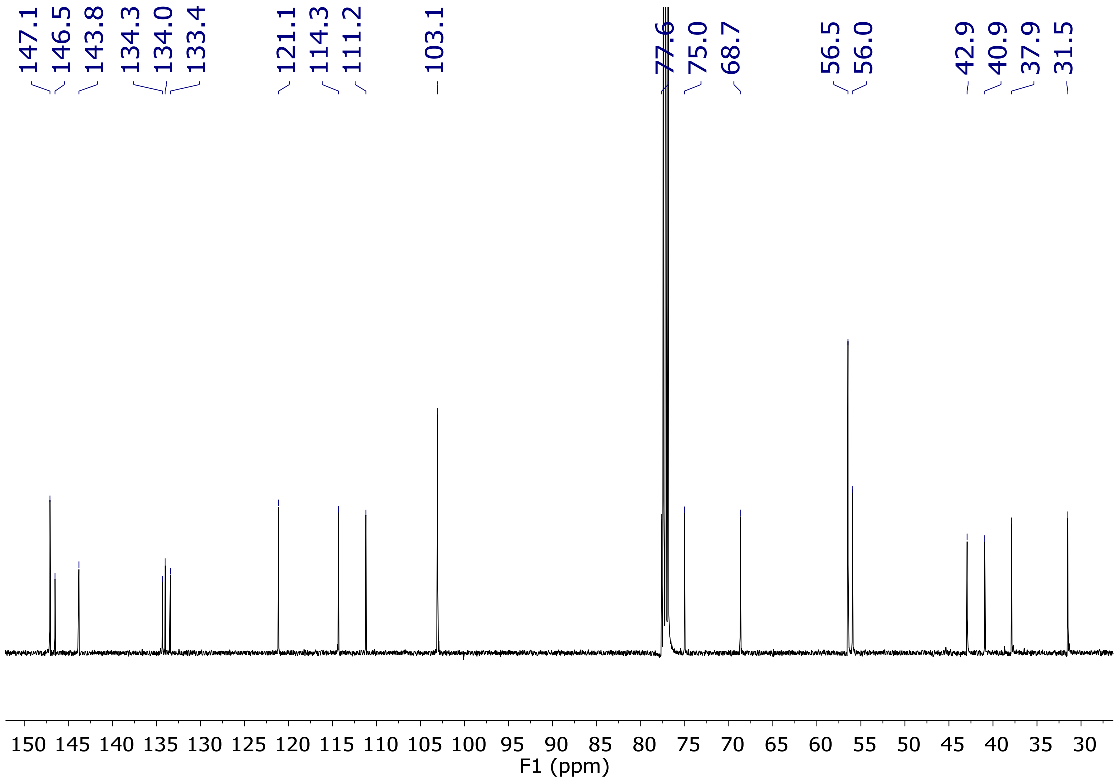

Fig. S10 ^{13}C NMR spectrum of compound **2**.

Acquisition Parameter

Source Type	ESI	Ion Polarity	Positive	Set Nebulizer	0.4 Bar
Focus	Active	Set Capillary	4500 V	Set Dry Heater	180 °C
Scan Begin	100 m/z	Set End Plate Offset	-500 V	Set Dry Gas	4.0 l/min
Scan End	2000 m/z	Set Charging Voltage	0 V	Set Divert Valve	Waste
		Set Corona	0 nA	Set APCI Heater	0 °C

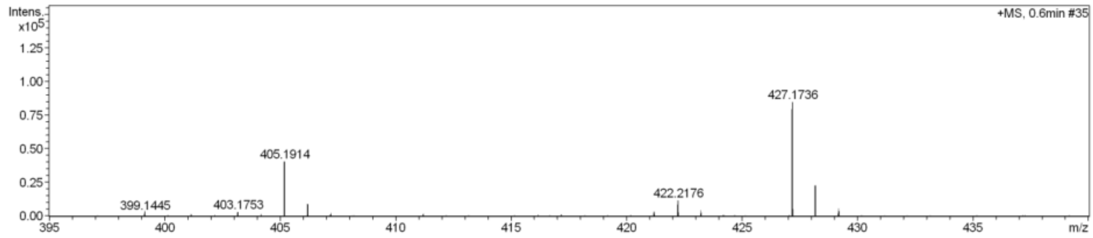

Meas. m/z	#	Ion Formula	Score	m/z	err [ppm]	err [mDa]	mSigma	rdb	e⁻ Conf	N-Rule
405.1914	1	C22H29O7	100.00	405.1908	-1.5	-0.6	11.0	8.5	even	ok
427.1736	1	C22H28NaO7	100.00	427.1727	2.0	0.8	14.6	8.5	even	ok
809.3747	1	C44H57O14	100.00	809.3743	0.5	0.4	78.4	16.5	even	ok
831.3576	1	C44H56NaO14	100.00	831.3562	1.7	1.4	13.2	16.5	even	ok

Fig. S11 HRESI-MS spectrum of compound **2**.

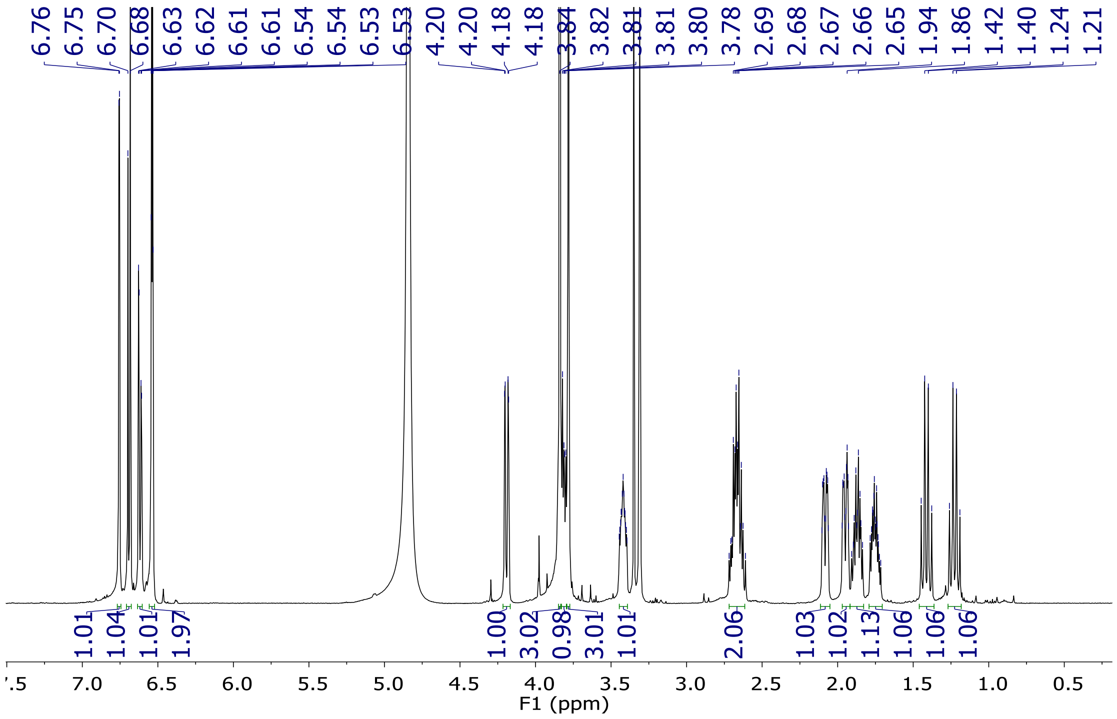

Fig. S12 ^{1}H NMR spectrum of compound **3**.

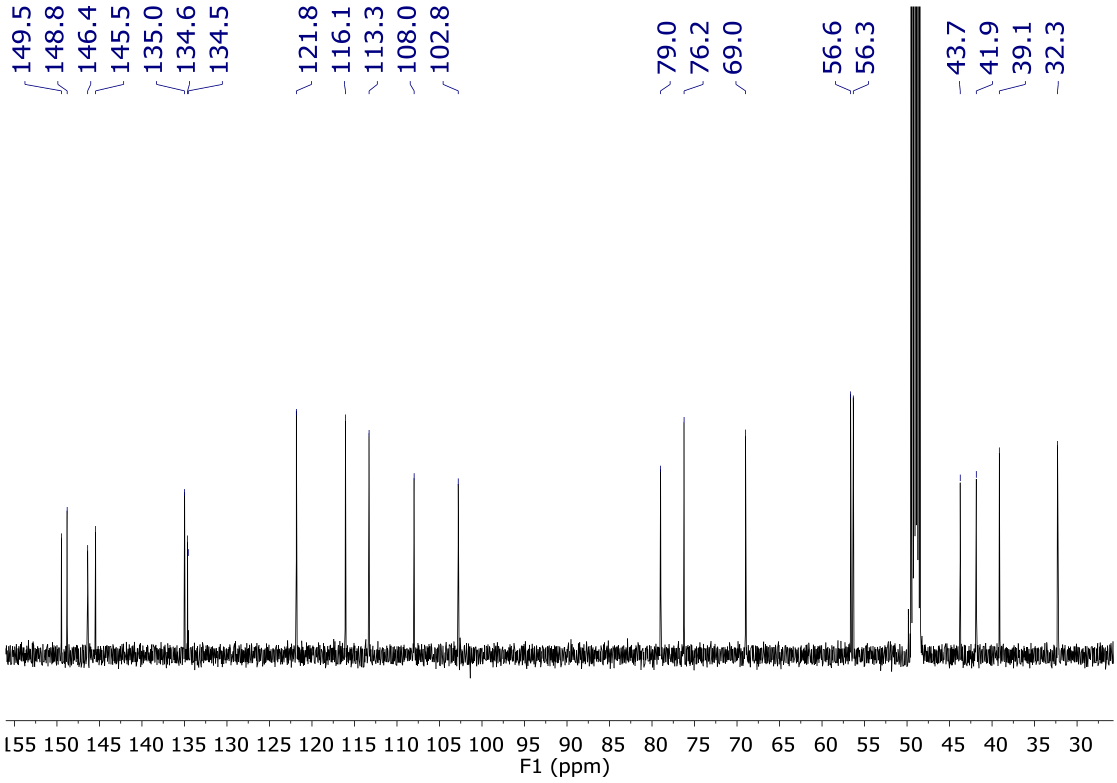

Fig. S13 ^{13}C NMR spectrum of compound **3**.

Acquisition Parameter

Source Type	ESI	Ion Polarity	Positive	Set Nebulizer	0.4 Bar
Focus	Active	Set Capillary	4500 V	Set Dry Heater	180 °C
Scan Begin	100 m/z	Set End Plate Offset	-500 V	Set Dry Gas	4.0 l/min
Scan End	2000 m/z	Set Charging Voltage	0 V	Set Divert Valve	Waste
		Set Corona	0 nA	Set APCI Heater	0 °C

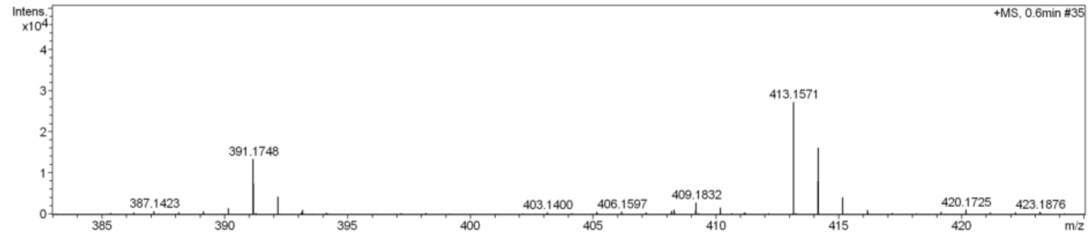

Meas. m/z	#	Ion Formula	Score	m/z	err [ppm]	err [mDa]	mSigma	rdb	e¯ Conf	N-Rule
391.1748	1	C21H27O7	100.00	391.1751	-0.8	-0.3	47.6	8.5	even	ok
413.1571	1	C21H26NaO7	100.00	413.1571	0.0	0.0	186.6	8.5	even	ok
781.3411	1	C42H53O14	100.00	781.3430	2.4	1.8	197.0	16.5	even	ok
803.3242	1	C42H52NaO14	100.00	803.3249	0.9	0.7	361.5	16.5	even	ok

Fig. S14 HRESI-MS spectrum of compound **3**.

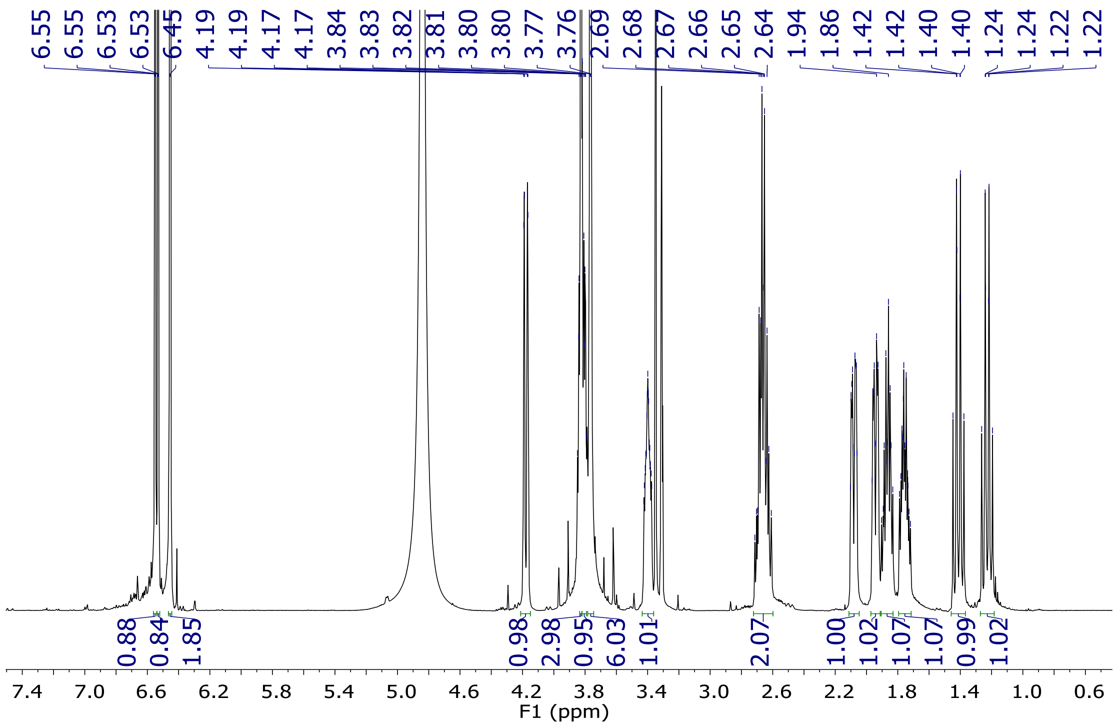

Fig. S15 ^{1}H NMR spectrum of compound **4**.

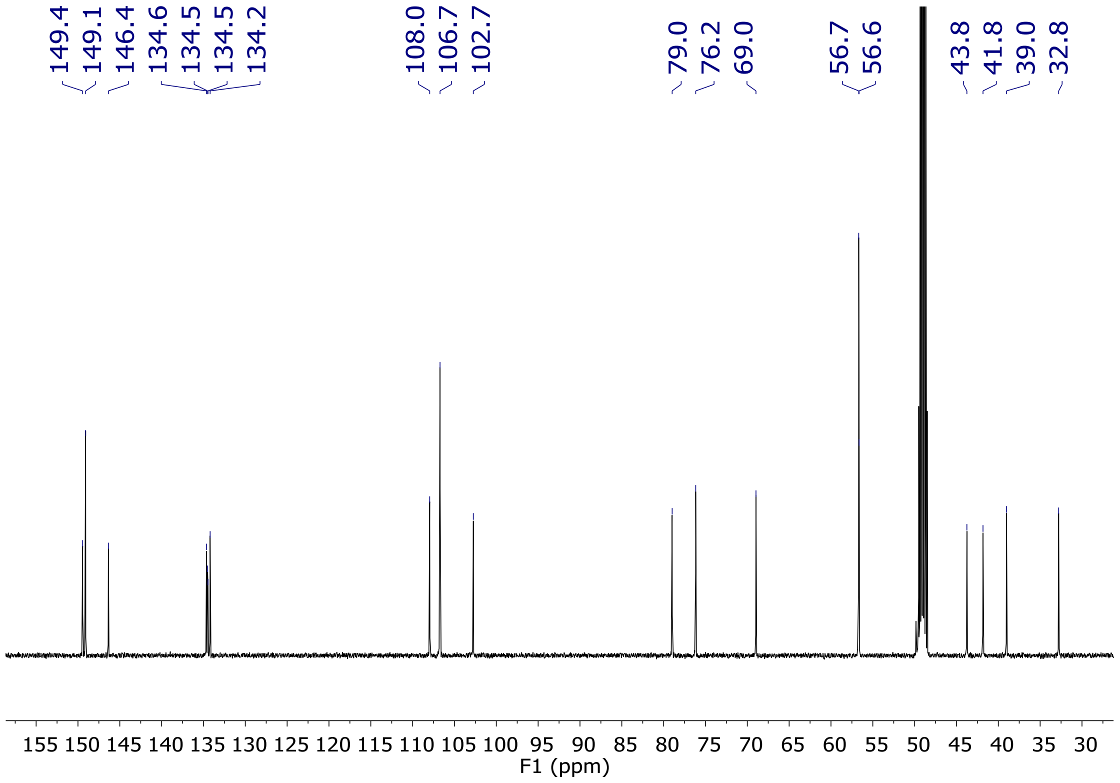

Fig. S16 ^{13}C NMR spectrum of compound **4**.

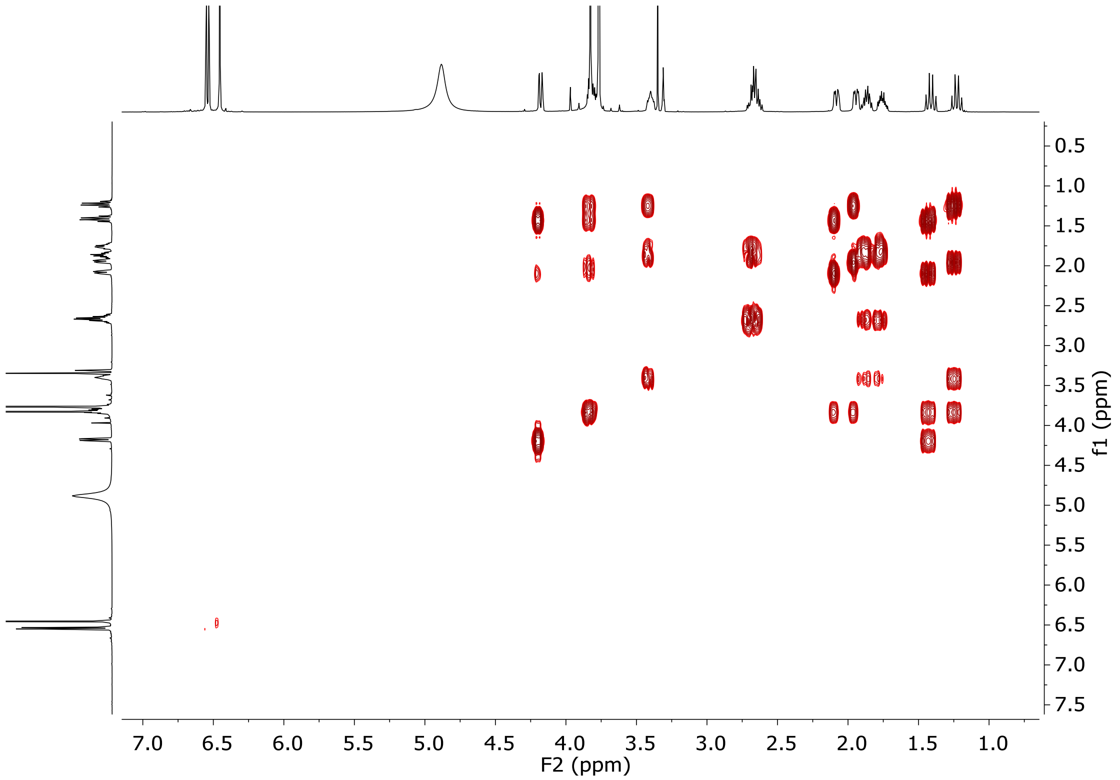

Fig. S17 ^{1}H–^{1}H COSY spectrum of compound **4**.

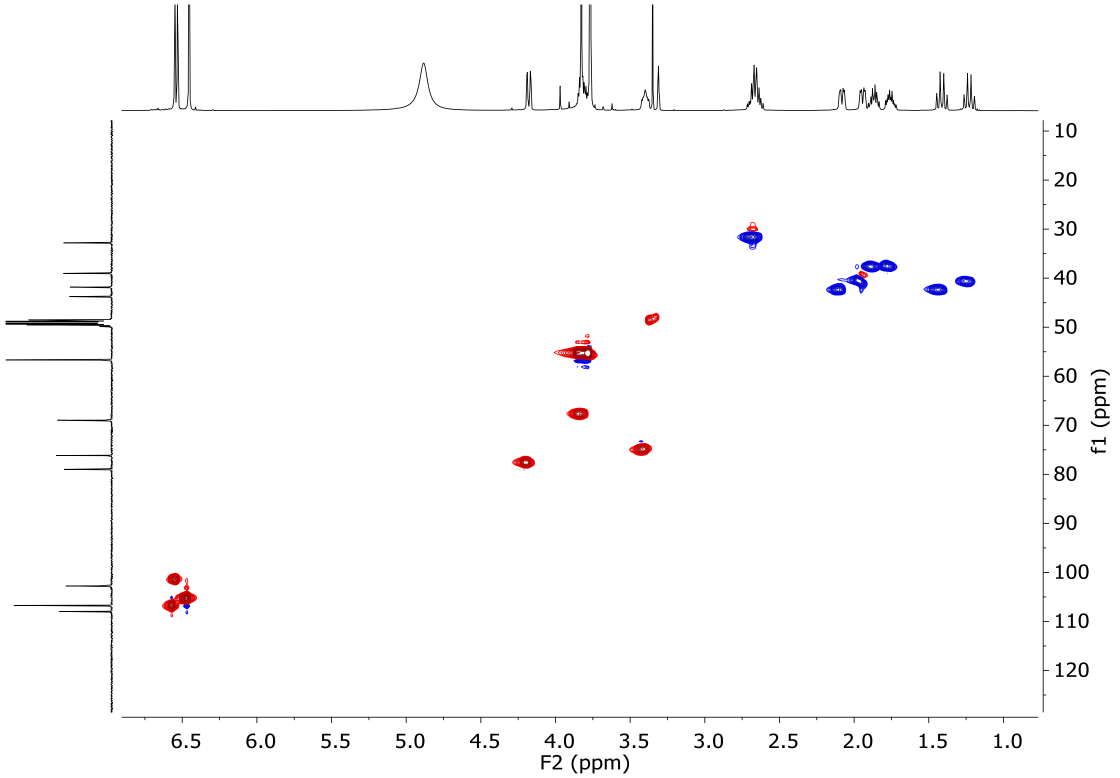

Fig. S18 HSQC spectrum of compound **4**.

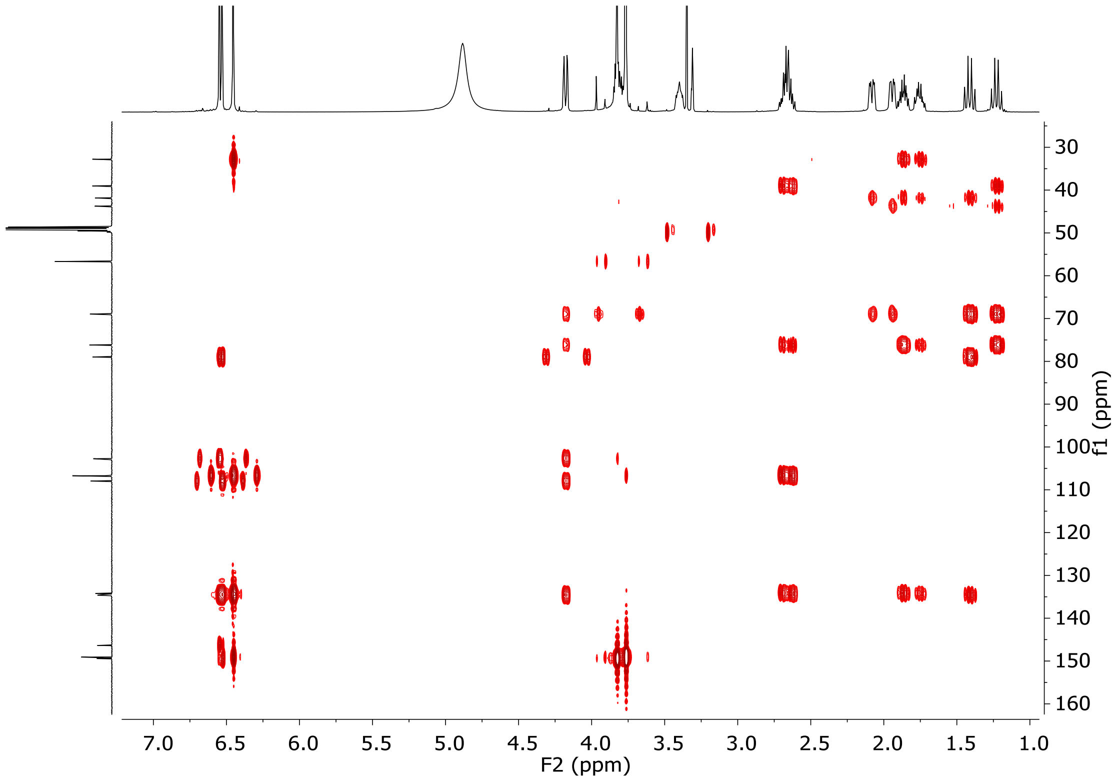

Fig. S19 HMBC spectrum of compound **4**.

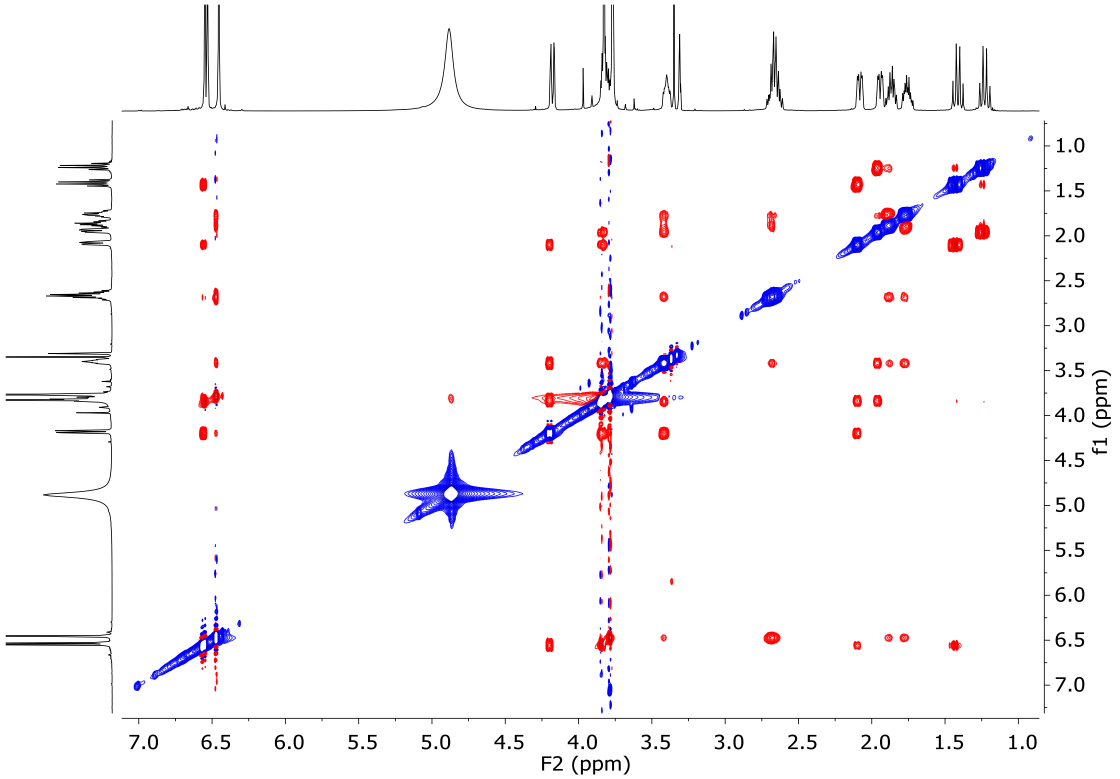

Fig. S20 NOESY spectrum of compound **4**.

Acquisition Parameter

Source Type	ESI	Ion Polarity	Positive	Set Nebulizer	0.4 Bar
Focus	Active	Set Capillary	4500 V	Set Dry Heater	180 °C
Scan Begin	100 m/z	Set End Plate Offset	-500 V	Set Dry Gas	4.0 l/min
Scan End	2000 m/z	Set Charging Voltage	0 V	Set Divert Valve	Waste
		Set Corona	0 nA	Set APCI Heater	0 °C

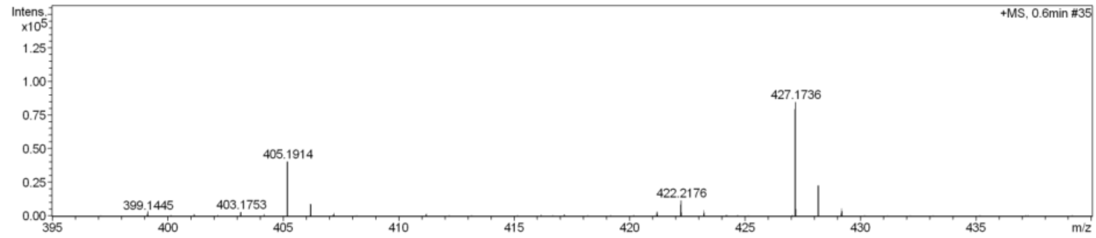

Meas. m/z	#	Ion Formula	Score	m/z	err [ppm]	err [mDa]	mSigma	rdb	e⁻ Conf	N-Rule
405.1914	1	C22H29O7	100.00	405.1908	-1.5	-0.6	11.0	8.5	even	ok
427.1736	1	C22H28NaO7	100.00	427.1727	2.0	0.8	14.6	8.5	even	ok
809.3747	1	C44H57O14	100.00	809.3743	0.5	0.4	78.4	16.5	even	ok
831.3576	1	C44H56NaO14	100.00	831.3562	1.7	1.4	13.2	16.5	even	ok

Fig. S21 HRESI-MS spectrum of compound **4**.

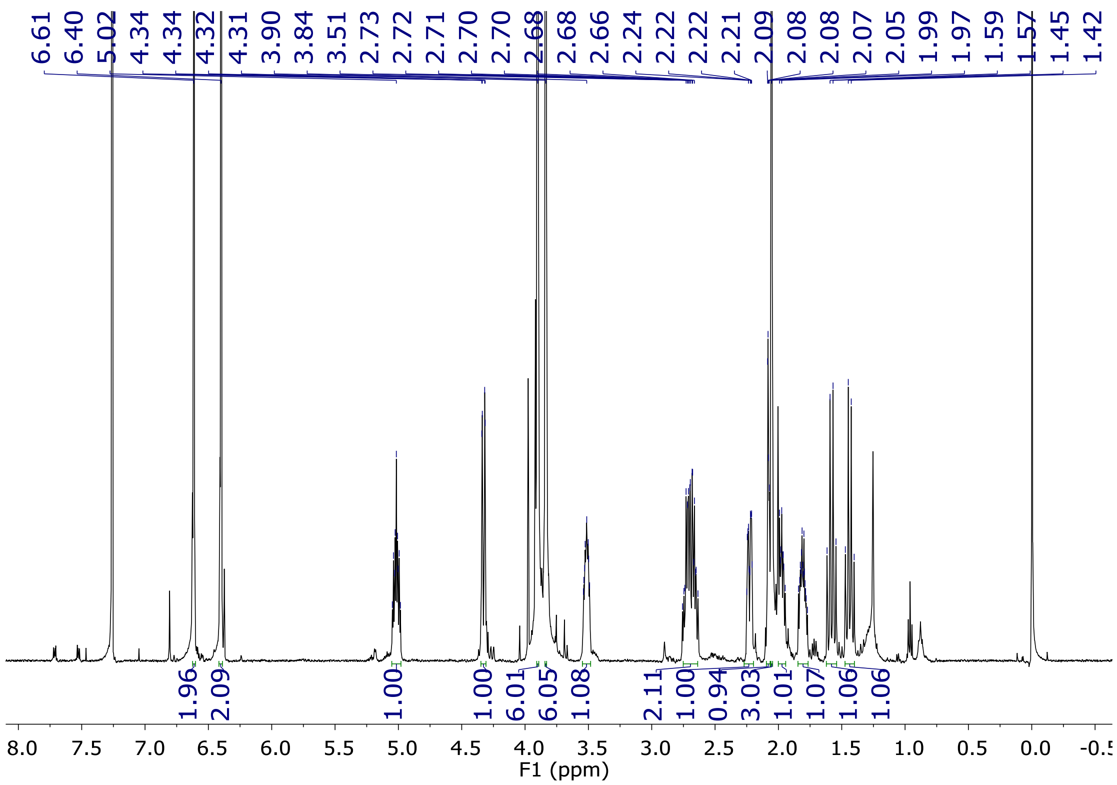

Fig. S22 ^{1}H NMR spectrum of compound **5**.

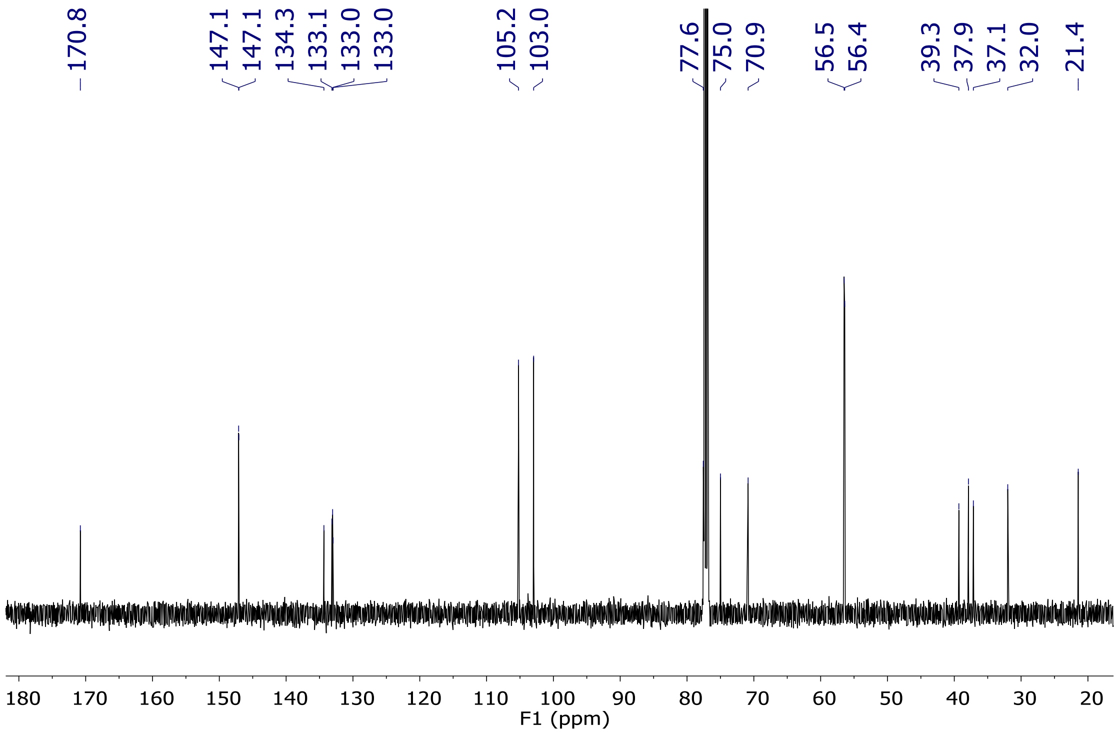

Fig. S23 ^{13}C NMR spectrum of compound **5**.

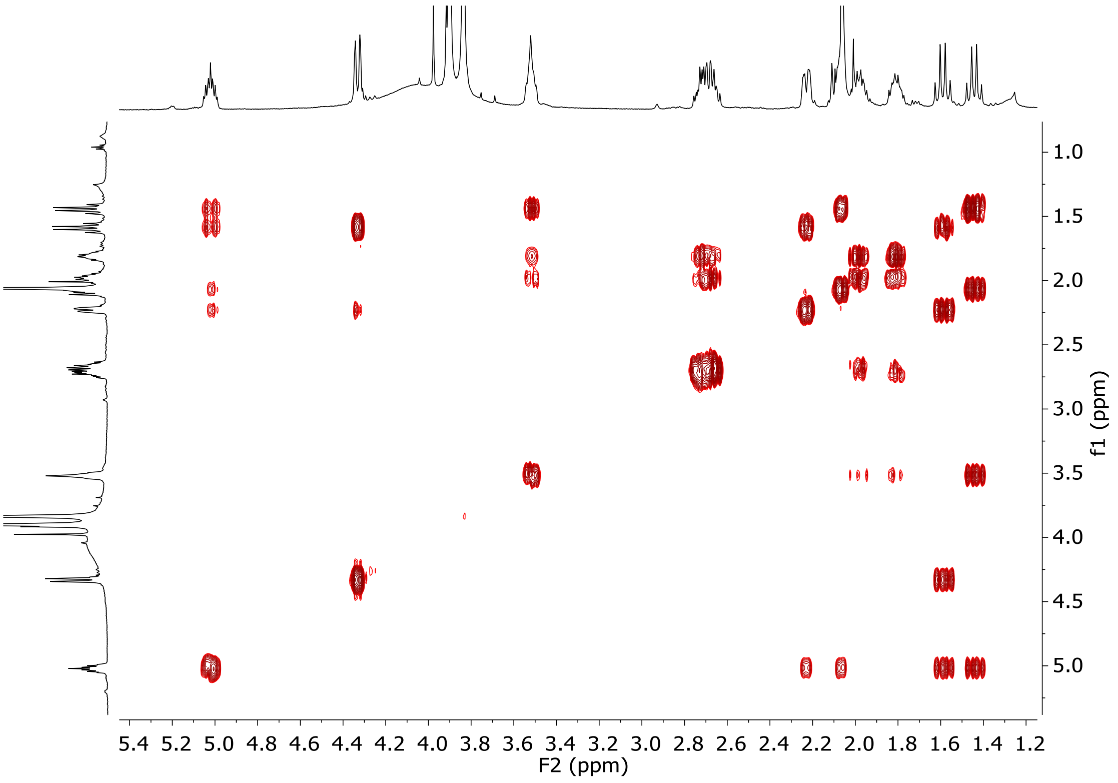

Fig. S24 ^{1}H–^{1}H COSY spectrum of compound **5**.

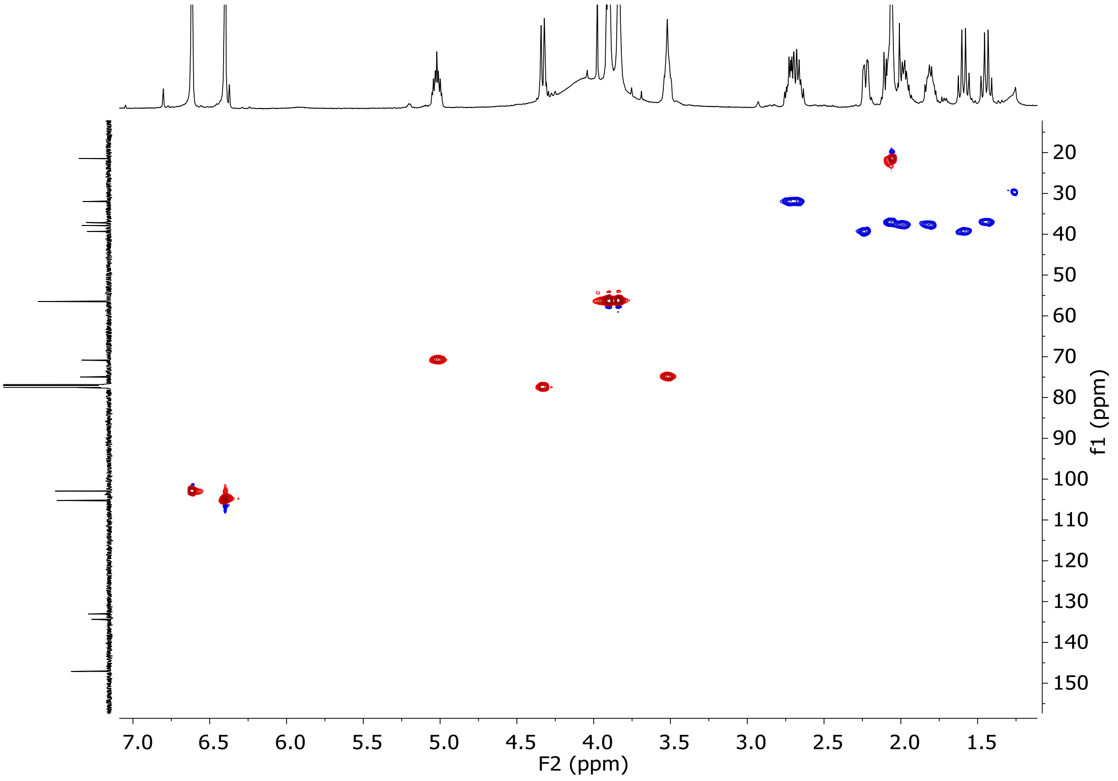

Fig. S25 HSQC spectrum of compound **5**.

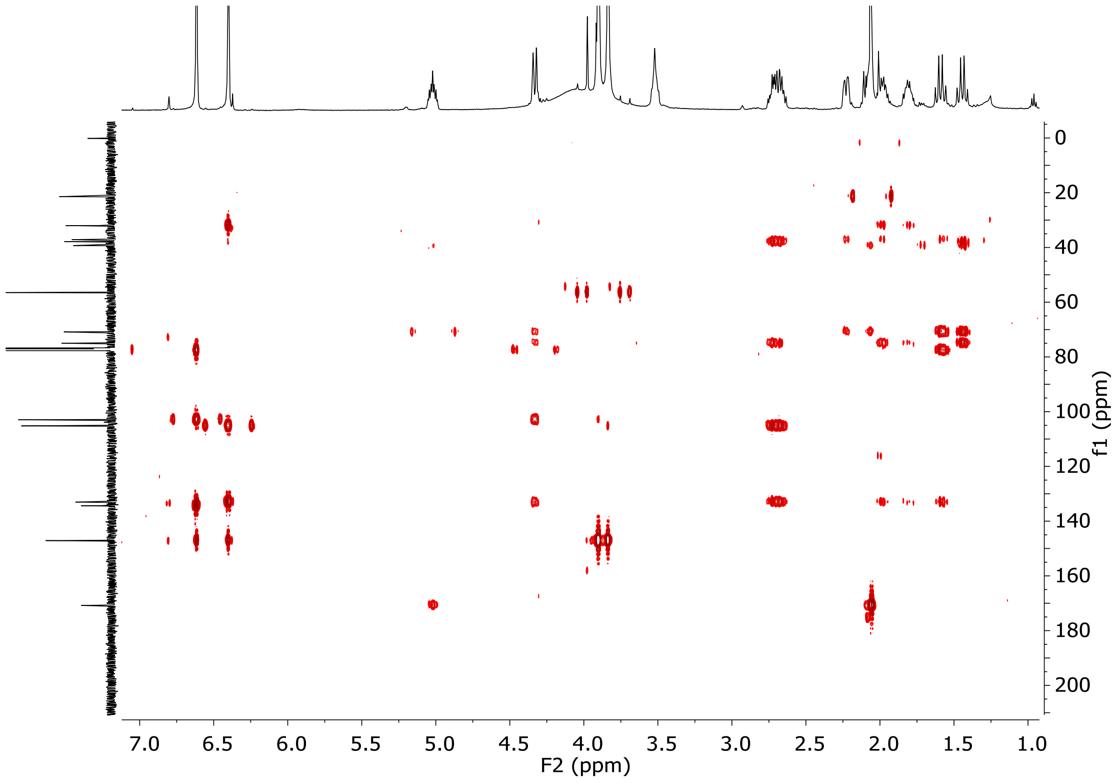

Fig. S26 HMBC spectrum of compound **5**.

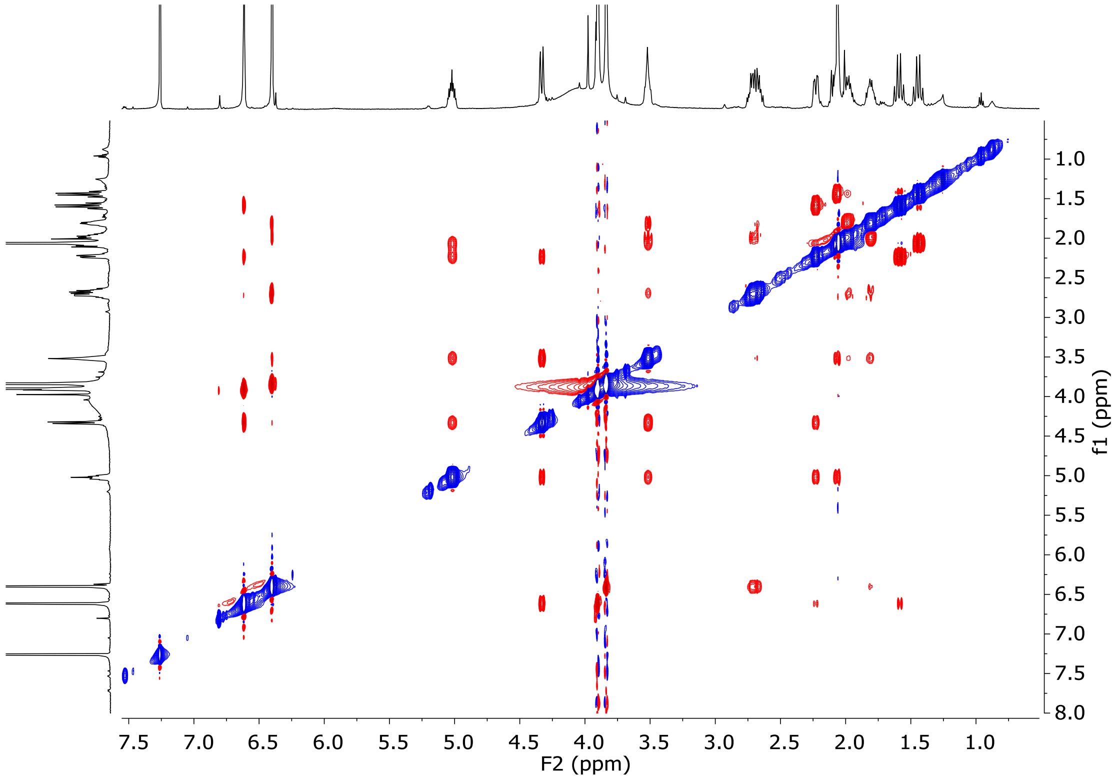

Fig. S27 NOESY spectrum of compound **5**.

Acquisition Parameter

Source Type	ESI	Ion Polarity	Positive	Set Nebulizer	0.4 Bar
Focus	Active	Set Capillary	4500 V	Set Dry Heater	180 °C
Scan Begin	100 m/z	Set End Plate Offset	-500 V	Set Dry Gas	4.0 l/min
Scan End	2000 m/z	Set Charging Voltage	0 V	Set Divert Valve	Waste
		Set Corona	0 nA	Set APCI Heater	0 °C

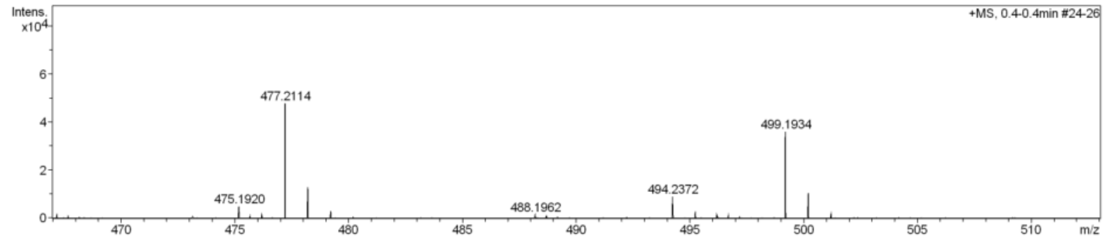

Meas. m/z	#	Ion Formula	Score	m/z	err [ppm]	err [mDa]	mSigma	rdb	e⁻ Conf	N-Rule
477.2114	1	C25H33O9	100.00	477.2119	-1.0	-0.5	4.7	9.5	even	ok
499.1934	1	C25H32NaO9	100.00	499.1939	1.0	0.5	7.9	9.5	even	ok
975.3976	1	C50H64NaO18	100.00	975.3985	-0.9	-0.9	15.2	18.5	even	ok

Fig. S28 HRESI-MS spectrum of compound **5**.

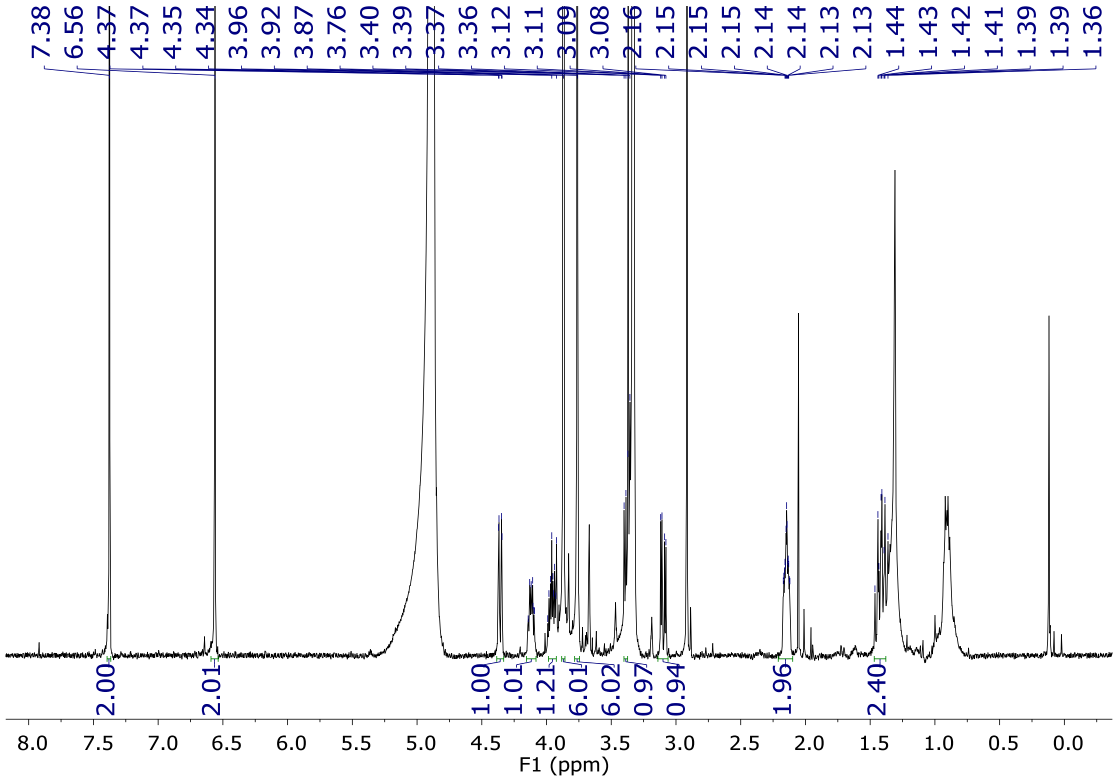

Fig. S29 ^{1}H NMR spectrum of compound **6**.

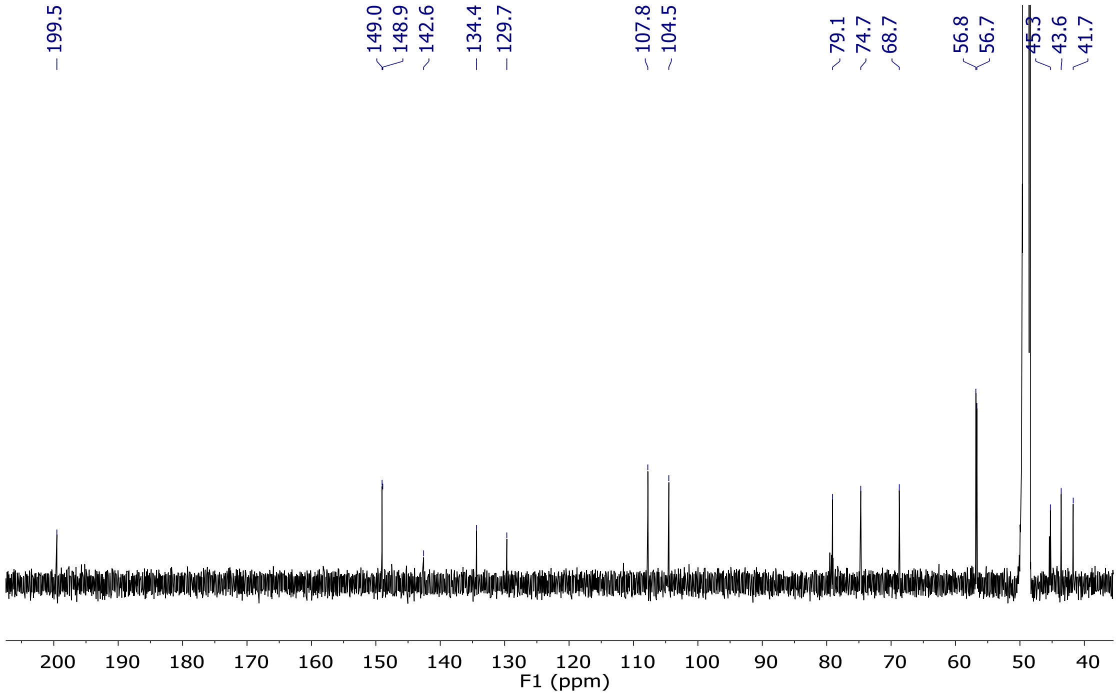

Fig. S30 ^{13}C NMR spectrum of compound **6**.

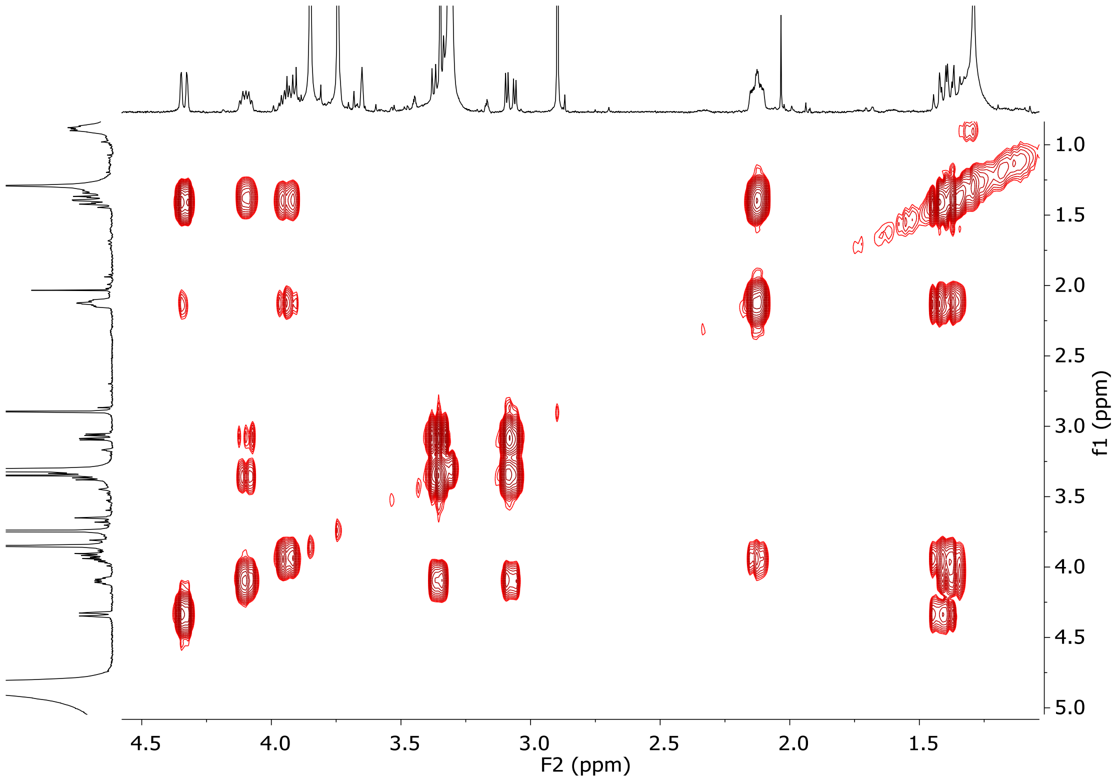

Fig. S31 ^{1}H–^{1}H COSY spectrum of compound **6**.

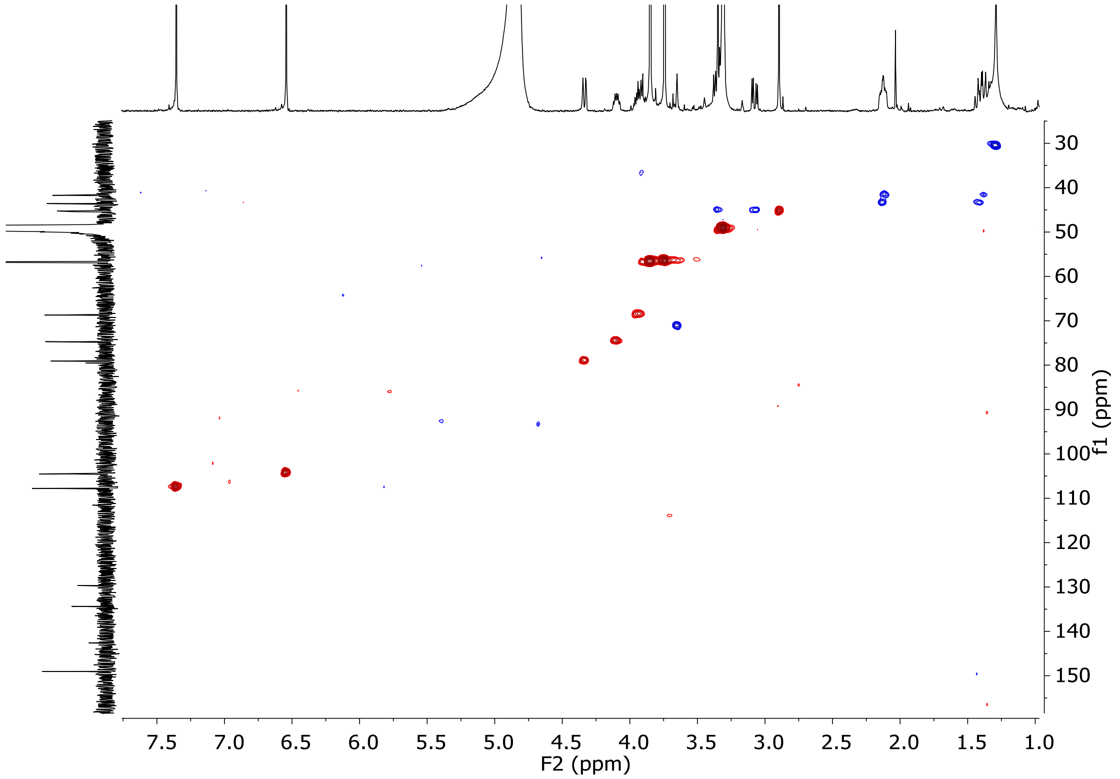

Fig. S32 HSQC spectrum of compound **6**.

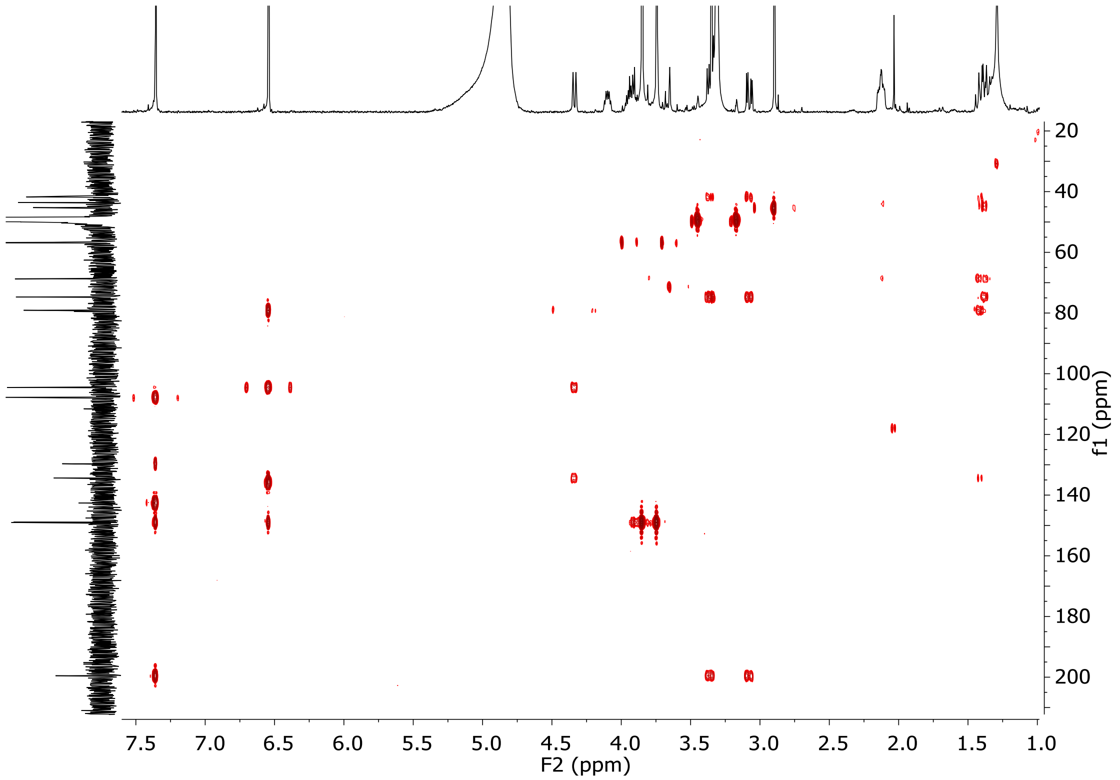

Fig. S33 HMBC spectrum of compound **6**.

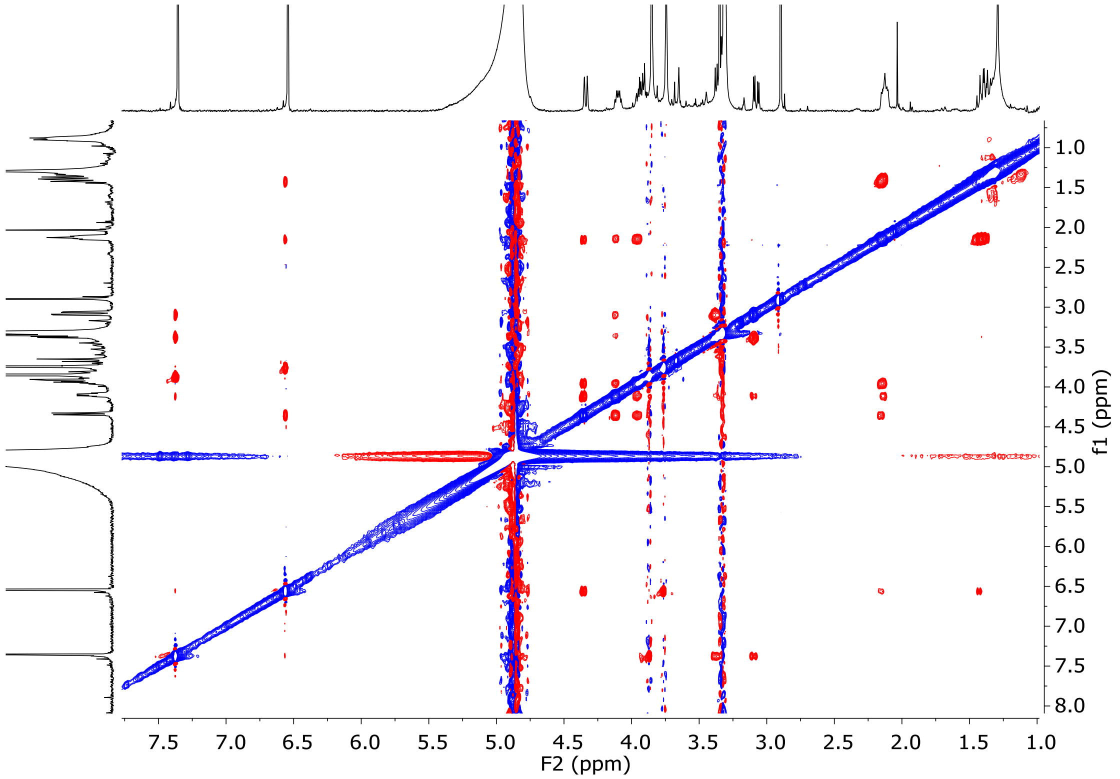

Fig. S34 NOESY spectrum of compound **6**.

Acquisition Parameter

Source Type	ESI	Ion Polarity	Positive	Set Nebulizer	0.4 Bar
Focus	Active	Set Capillary	4500 V	Set Dry Heater	180 °C
Scan Begin	100 m/z	Set End Plate Offset	-500 V	Set Dry Gas	4.0 l/min
Scan End	2000 m/z	Set Charging Voltage	0 V	Set Divert Valve	Waste
		Set Corona	0 nA	Set APCI Heater	0 °C

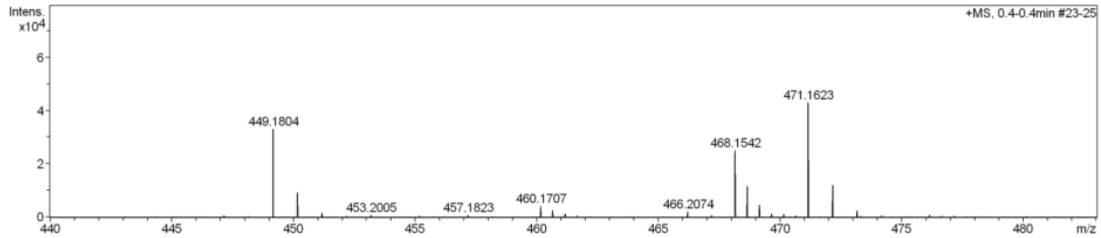

Meas. m/z	#	Ion Formula	Score	m/z	err [ppm]	err [mDa]	mSigma	rdb	e⁻ Conf	N-Rule
449.1804	1	$C_{23}H_{29}O_9$	100.00	449.1806	0.4	0.2	12.3	9.5	even	ok
471.1623	1	$C_{23}H_{28}NaO_9$	100.00	471.1626	0.5	0.2	14.5	9.5	even	ok
897.3545	1	$C_{46}H_{57}O_{18}$	100.00	897.3539	0.6	0.6	80.6	18.5	even	ok
919.3356	1	$C_{46}H_{56}NaO_{18}$	100.00	919.3359	0.4	0.3	33.5	18.5	even	ok

Fig. S35 HRESI-MS spectrum of compound **6**.

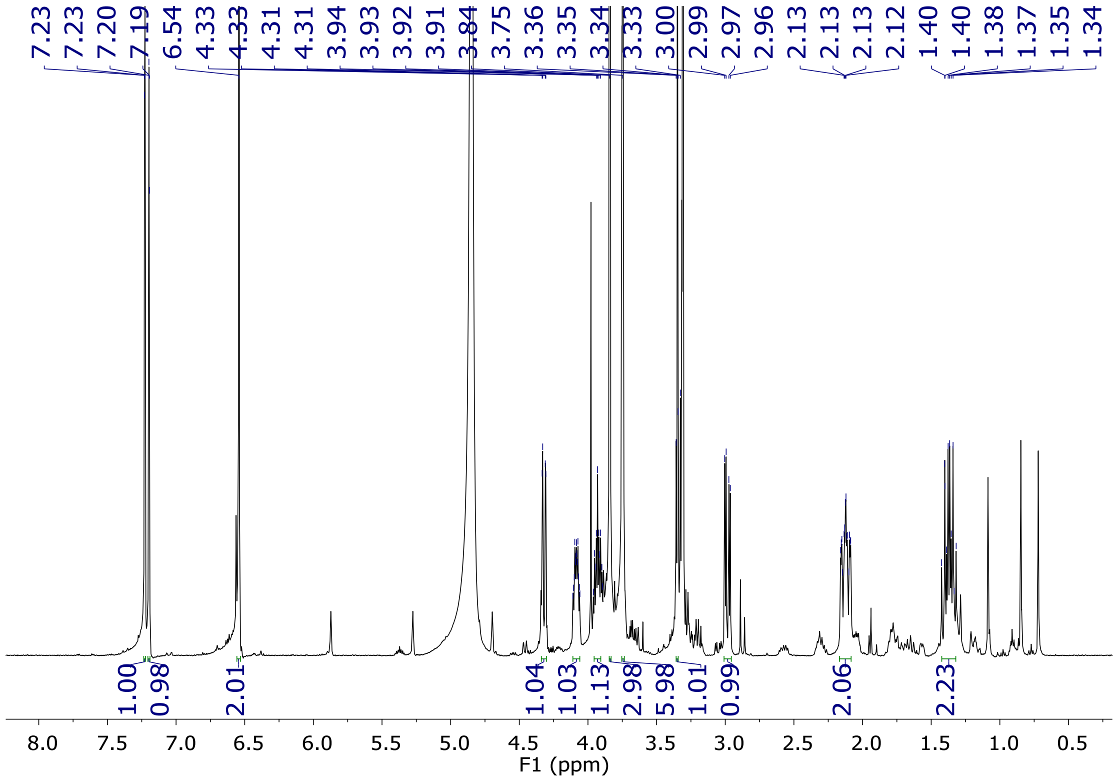

Fig. S36 ^{1}H NMR spectrum of compound **7**.

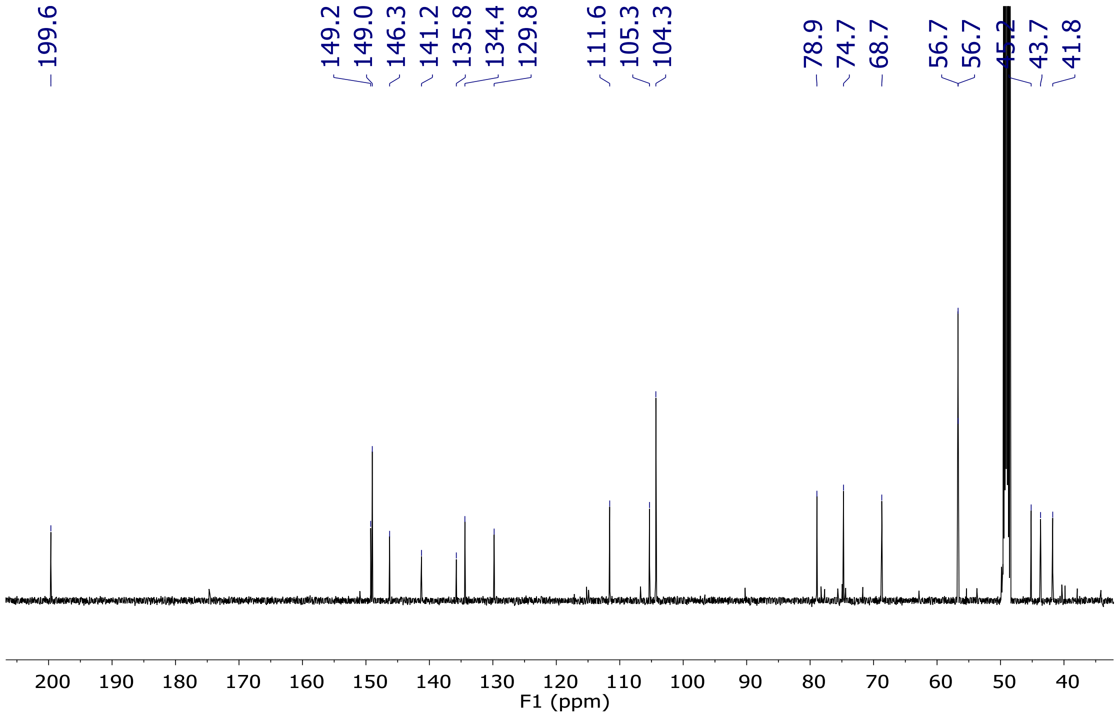

Fig. S37 ^{13}C NMR spectrum of compound **7**.

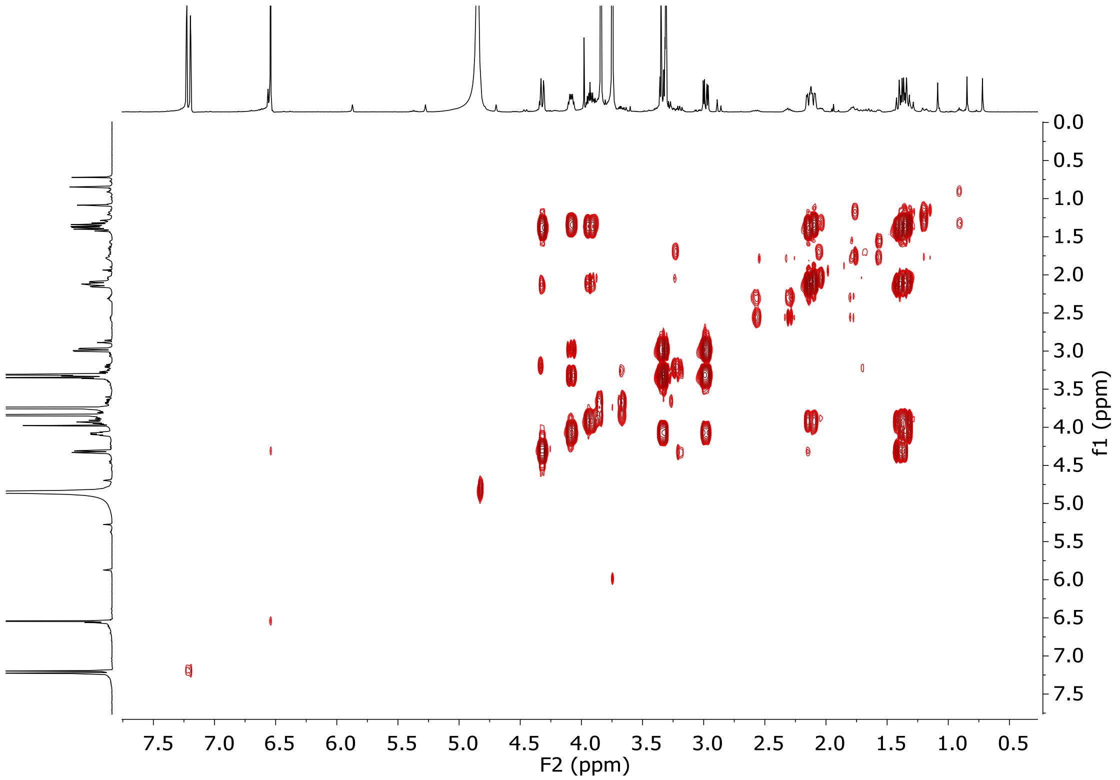

Fig. S38 ^{1}H–^{1}H COSY spectrum of compound **7**.

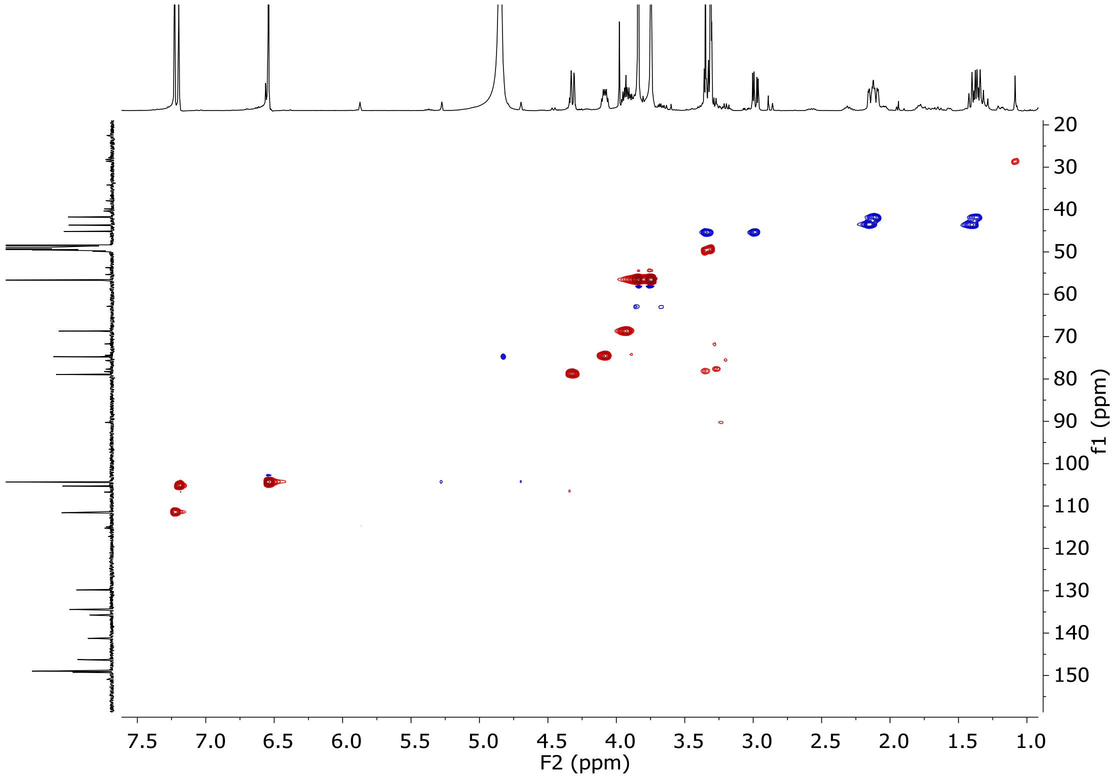

Fig. S39 HSQC spectrum of compound **7**.

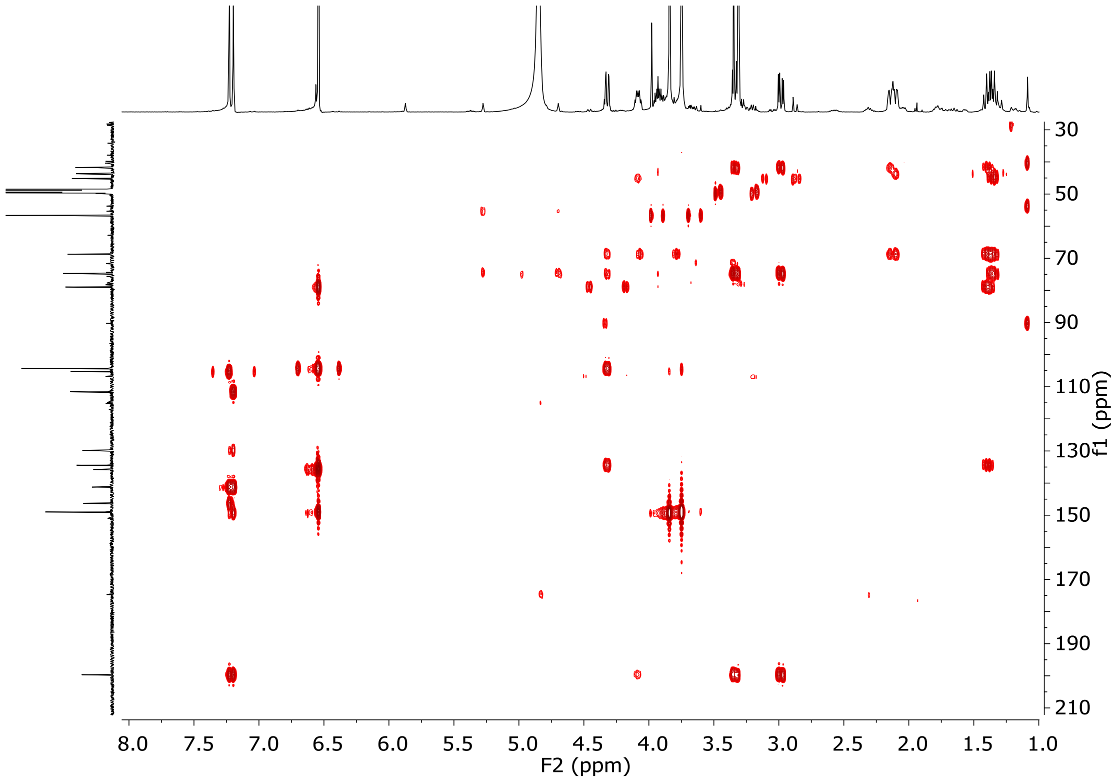

Fig. S40 HMBC spectrum of compound **7**.

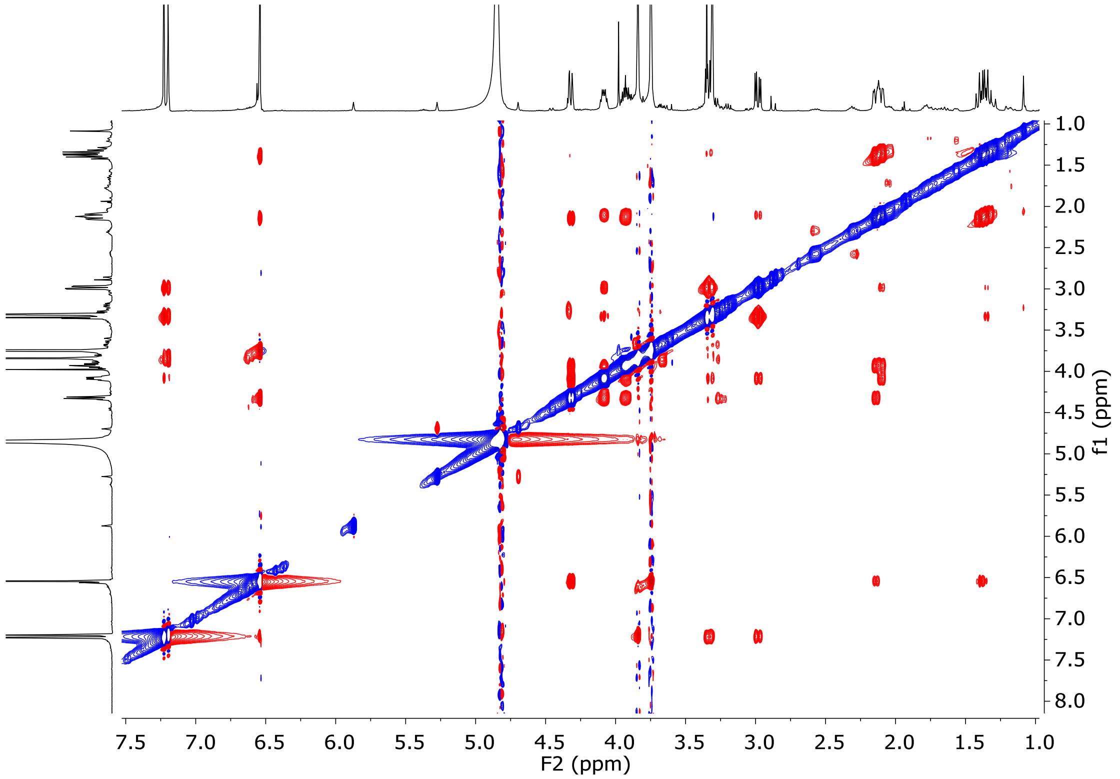

Fig. S41 NOESY spectrum of compound **7**.

Acquisition Parameter

Source Type	ESI	Ion Polarity	Positive	Set Nebulizer	0.4 Bar
Focus	Active	Set Capillary	4500 V	Set Dry Heater	180 °C
Scan Begin	100 m/z	Set End Plate Offset	-500 V	Set Dry Gas	4.0 l/min
Scan End	2000 m/z	Set Charging Voltage	0 V	Set Divert Valve	Waste
		Set Corona	0 nA	Set APCI Heater	0 °C

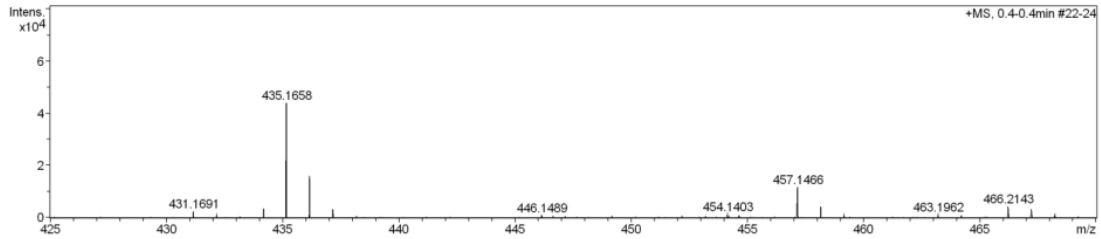

Meas. m/z	#	Ion Formula	Score	m/z	err [ppm]	err [mDa]	mSigma	rdb	e⁻ Conf	N-Rule
435.1658	1	C22H27O9	100.00	435.1650	-1.9	-0.8	63.0	9.5	even	ok
457.1466	1	C22H26NaO9	100.00	457.1469	0.6	0.3	60.1	9.5	even	ok
869.3237	1	C44H53O18	100.00	869.3226	-1.2	-1.1	104.8	18.5	even	ok
891.3044	1	C44H52NaO18	100.00	891.3046	0.2	0.2	95.7	18.5	even	ok

Fig. S42 HRESI-MS spectrum of compound **7**.

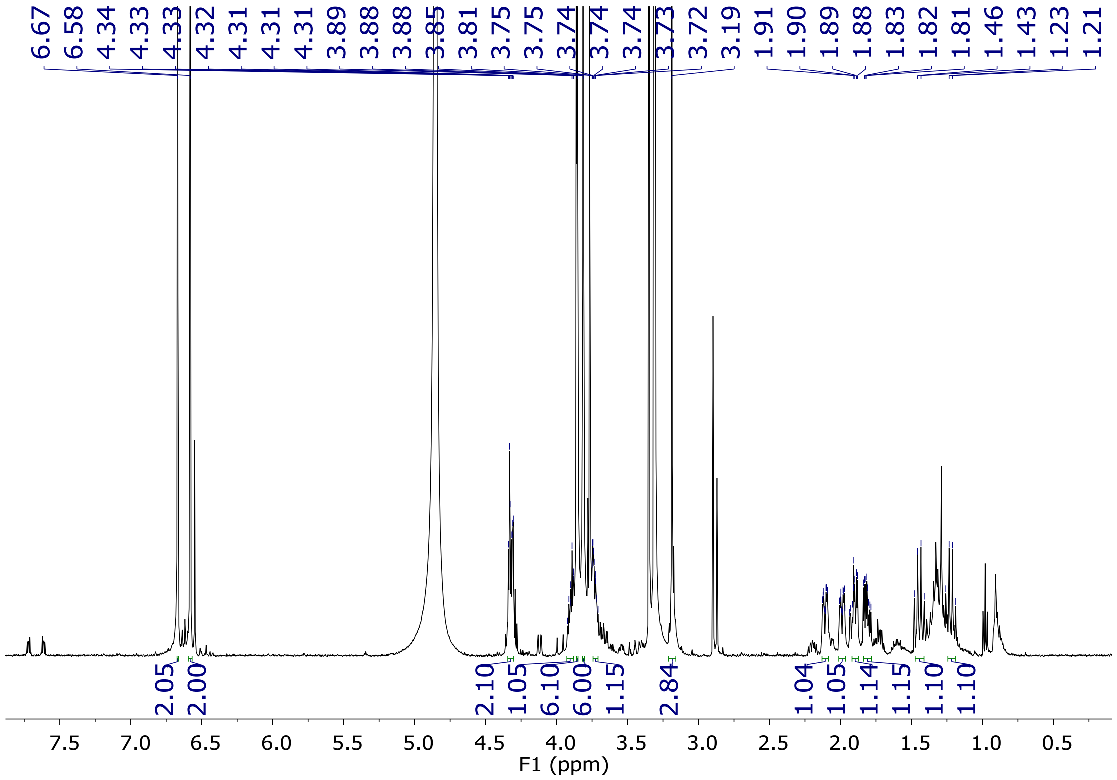

Fig. S43 ^{1}H NMR spectrum of compound **8**.

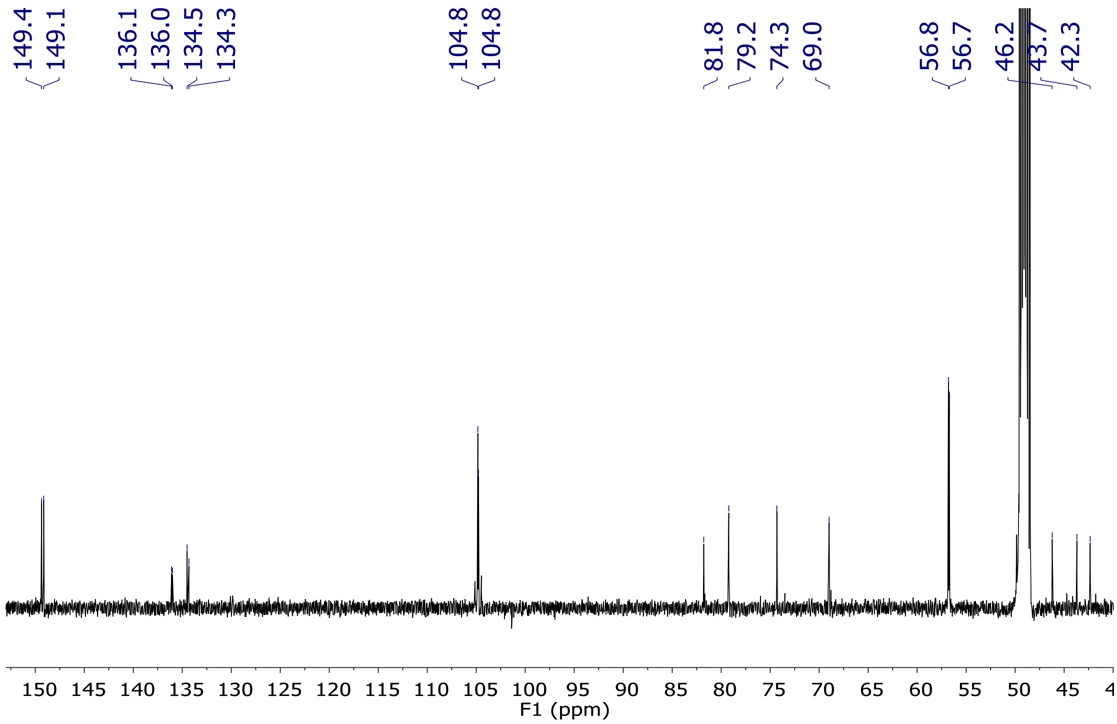

Fig. S44 ^{13}C NMR spectrum of compound **8**.

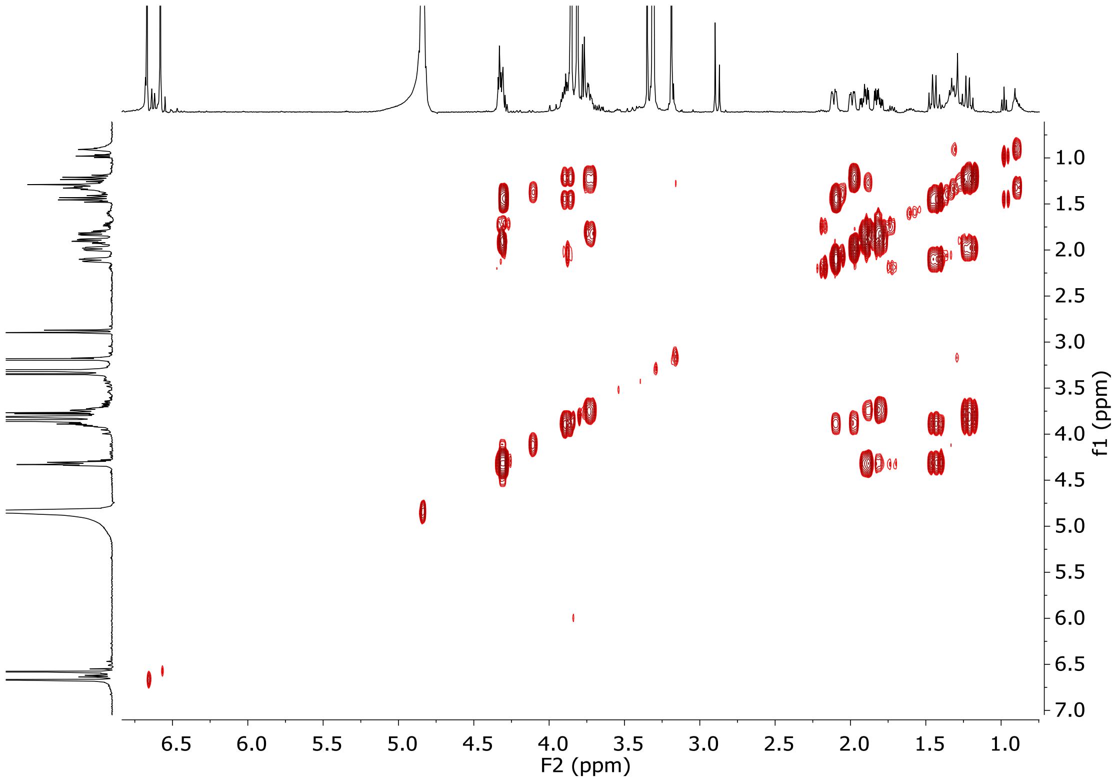

Fig. S45 $^{1}H–^{1}H$ COSY spectrum of compound **8**.

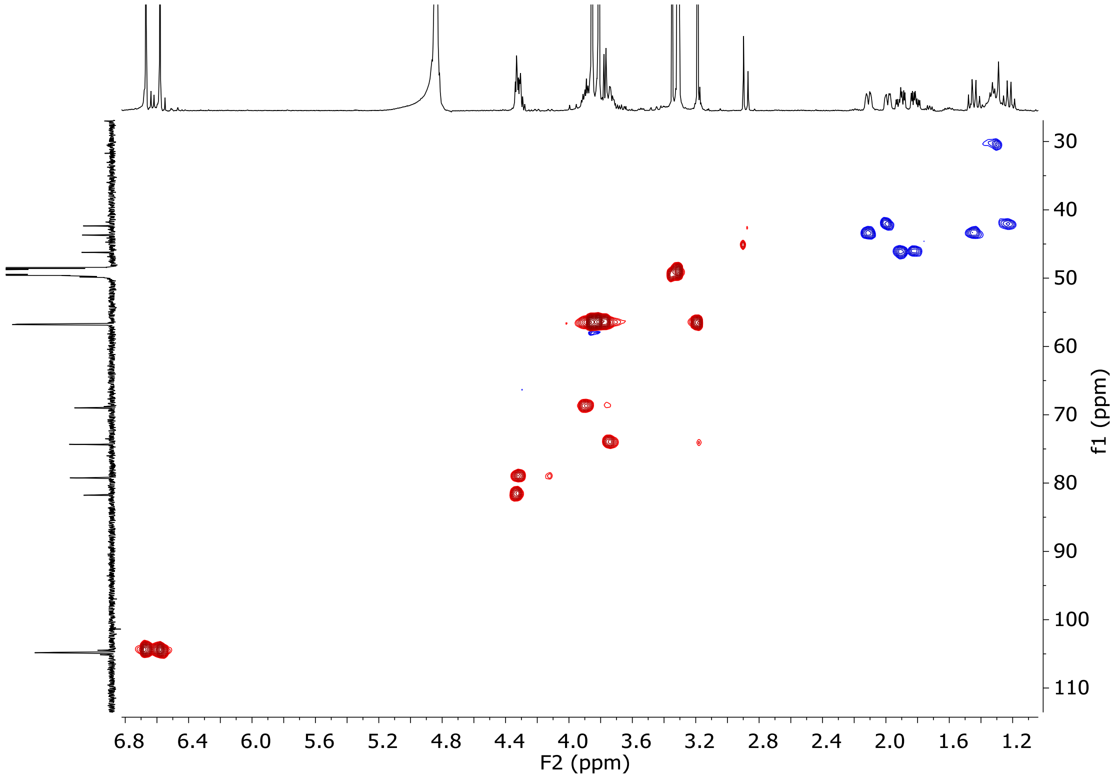

Fig. S46 HSQC spectrum of compound **8**.

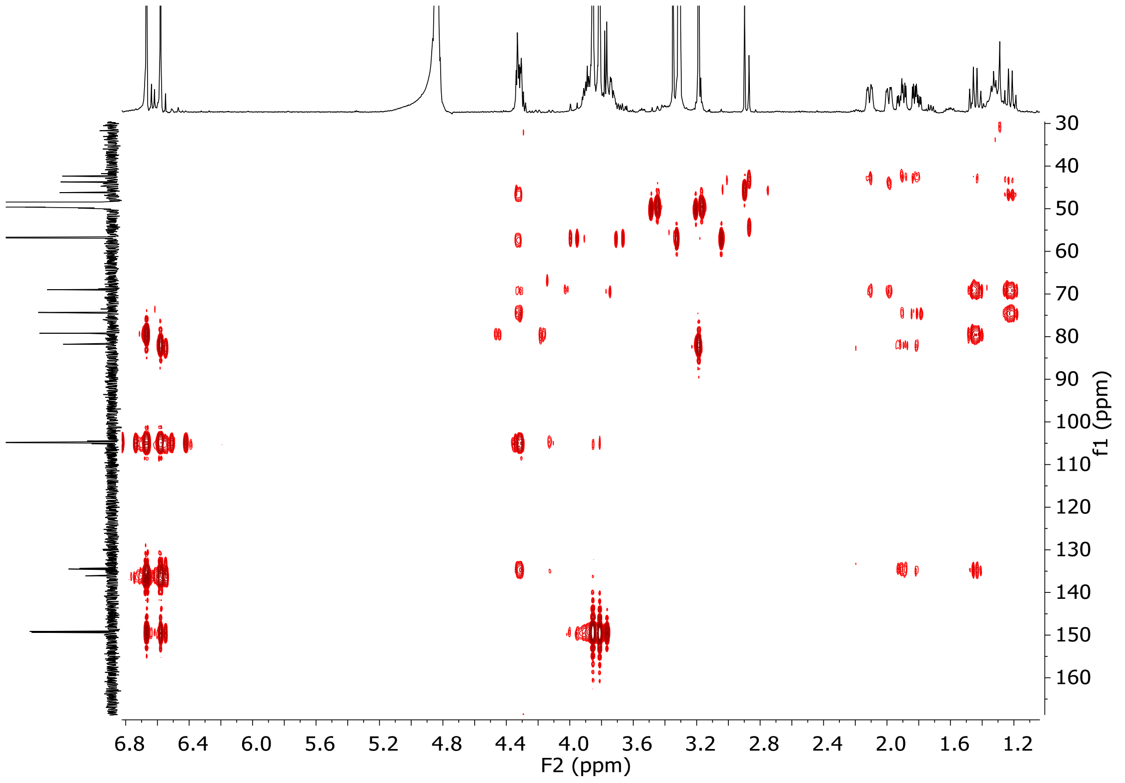

Fig. S47 HMBC spectrum of compound **8**.

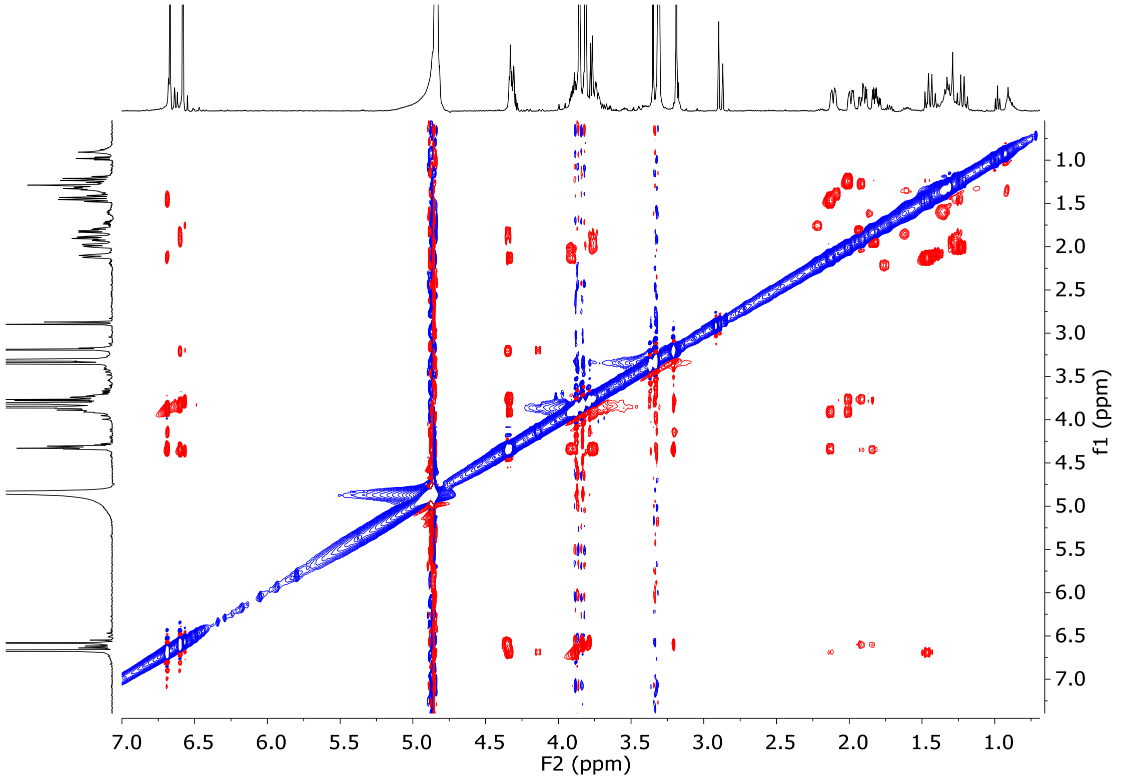

Fig. S48 NOESY spectrum of compound **8**.

Acquisition Parameter

Source Type	ESI	Ion Polarity	Positive	Set Nebulizer	0.4 Bar
Focus	Active	Set Capillary	4500 V	Set Dry Heater	180 °C
Scan Begin	100 m/z	Set End Plate Offset	-500 V	Set Dry Gas	4.0 l/min
Scan End	2000 m/z	Set Charging Voltage	0 V	Set Divert Valve	Waste
		Set Corona	0 nA	Set APCI Heater	0 °C

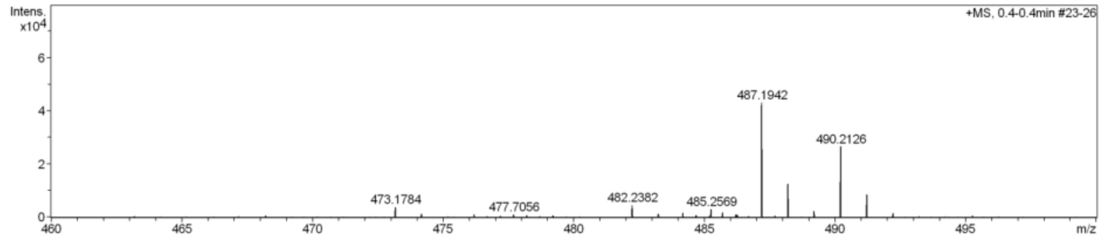

Meas. m/z	#	Ion Formula	Score	m/z	err [ppm]	err [mDa]	mSigma	rdb	e⁻ Conf	N-Rule
487.1942	1	C24H32NaO9	100.00	487.1939	-0.7	-0.3	306.2	8.5	even	ok
951.3994	1	C48H64NaO18	100.00	951.3985	-1.0	-0.9	505.3	16.5	even	ok

Fig. S49 HRESI-MS spectrum of compound **8**.
